# Enhancement and Machine Learning-Based Prediction of Tribological Properties of PC/PBT/GNPs Nanocomposites

**DOI:** 10.1021/acsomega.5c02538

**Published:** 2025-05-29

**Authors:** Tuba Özdemir Öge

**Affiliations:** 162311 Bartın University, Faculty of Forestry, Department of Forestry Industry Engineering, Ağdacı Campus, Bartin 74100, Turkey

## Abstract

Ternary polycarbonate-poly­(butylene terephthalate)/graphene nanoplatelets (PC–PBT/GNP) nanocomposites were fabricated by melt-compounding. The nanofiller dispersion, microstructural changes, and mechanical and tribological properties of the produced samples were investigated. The friction and wear performance of the produced samples were evaluated with a pin-on-disc test rig under 5 and 10 N loads against an AISI 52100 steel ball to evaluate the effect of GNP filler fraction on the friction and wear performance of PC–PBT blends subject to polymer–metal contact in automotive and aviation industries. The impact strength, tensile modulus, and flexural modulus of the neat PC–PBT blend were improved by 78, 46, and 38%, respectively, with the optimum nanofiller fraction of 5 wt %. In parallel to the improved mechanical properties, ∼86 and ∼90% reduction in specific wear rates were achieved under 5 and 10 N loads, respectively, compared to the neat sample, which is attributable to multiple factors such as increased stiffness contact surface, intrinsic lubricating characteristics of GNPs, a more tribo-layer-oriented wear regime at higher filler fractions, and increased crystallinity via the reduced extent of transesterification. The Least-Squares Boosting (LSBoost) machine learning model provided the highest prediction accuracy with *R* ^2^ = 0.9922 via incorporation of contact pressure calculation results into the model as dependent variables.

## Graphical abstract

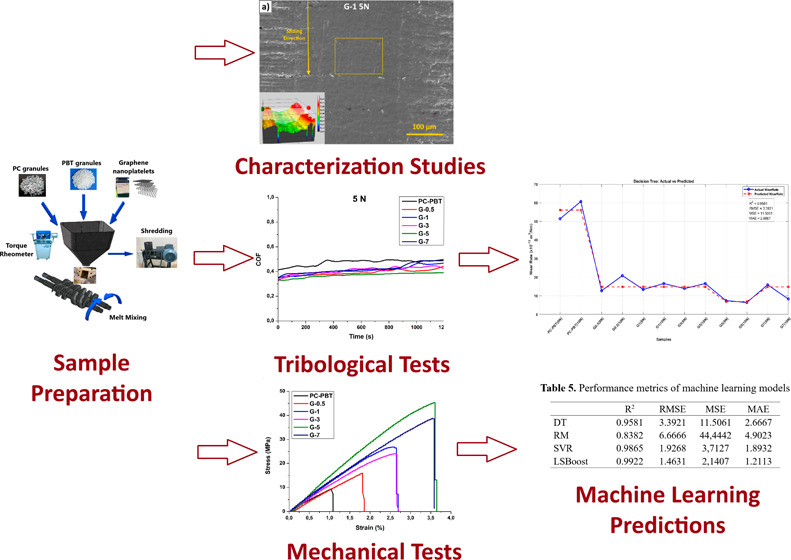

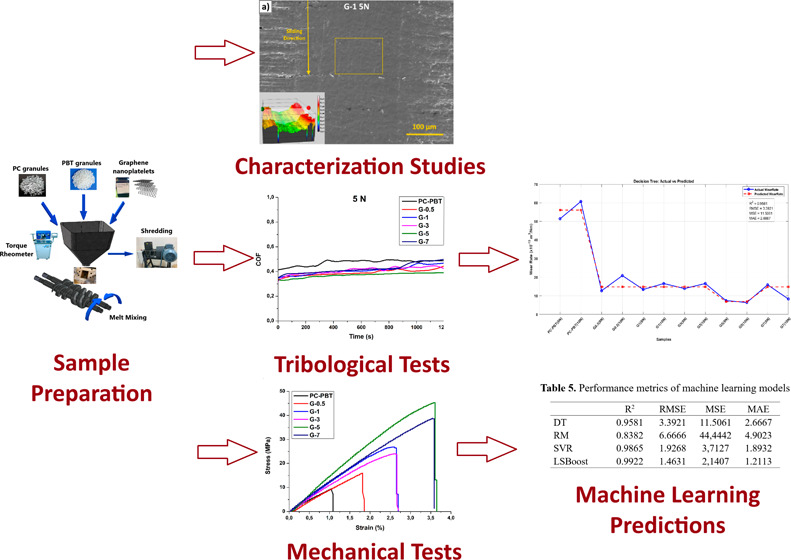

## 1 Introduction

Blending polymer matrices has been an effective method to extend the application fields of polymers by ameliorating the limitations of each component by the superior properties of others. In this regard, poly­(butylene terephthalate) (PBT), a semicrystalline engineering thermoplastic with high chemical stability, tensile strength, machinability, and low melt viscosity^1–5^ has been often used to modify the poor solvent resistance and high melt viscosity of polycarbonate (PC),^6–8^ an amorphous multipurpose thermoplastic also having superior properties such as high impact strength, rigidity, flame retardancy and operating temperature range in addition to its optical properties.^9–11^ PC–PBT blends thus stand out as one of the most widely commercialized polymer blends for various applications in key industries such as automotive and aviation.^10,12^ One of the most encountered issues in polymer blends produced via melt-compounding is the poor interaction at the interface of components resulting in poor miscibility and the low impact strength of the resultant blend^13–15^ which can be tackled by catalyzing the interaction between the constituent phases.^13,16^ In the case of PC/PBT blends, however, the main issue turns out to be the uncontrollable progress of transesterification reaction taking place between the molecular chains of the components under the shearing of melt-compounding, thus suppressing the crystallization behavior of PBT component and promoting the formation of random copolymers that impair the mechanical properties such as impact resistance.^5,16,17^ Consequently, to improve the impact resistance of PC–PBT blends, alternative routes have been followed involving the suppression of transesterification or incorporation of additional constituents such as elastomers, compatibilizers or nanofillers, as it has been reported that, the adverse effect of transesterification on crystallization of PC/PBT blends could be alleviated by the addition of nanofillers.^16^ CNTs as another form of carbon-based nanofillers have been reported to act as an effective nucleating agent to enhance crystallization via suppressing the mobility of polymer chains and forming heterogeneous nucleation sites, and this situation has been verified even with highly miscible blends where the crystallization ability of the semicrystalline constituent is largely suppressed^16,18^ beside the impact modifying capability of CNTs.^19^ Aside from acting as a nucleating agent, CNTs were reported to form a bridging effect among the blend constituents thus enhancing the stress transfer between them in the particular case of a percolated structure.^19^ Similar to CNTs, GNPs are also characterized by high aspect ratio and specific surface,^20^ thus they are expected to play a similar transesterification-alleviating effect, apart from their self-lubricating and stress-transferring capabilities.^21^ For PC–PBT blends, incorporation of nanofillers via melt-compounding emerges as a highly viable route not only for addressing the issue of excessive miscibility but also for drawing on the exceptional physical and chemical properties of nanofillers to enhance and complement several properties of the blend components. Moreover, the use of dry-blending methods such as melt-compounding requires the use of no solvents or chemical routes such as functionalization of nanofillers, thus providing certain environmental and cost-related advantages.^22–24^

In recent decades, reinforcing polymers with carbonaceous nanofillers such as carbon nanotubes (CNTs), graphene and their derivatives has been the subject of an increasing number of works due to the outstanding attributes of these materials such as high aspect ratio, modulus of elasticity, electrical and thermal conductivity aside from their electro-optical and electromagnetic interference shielding behaviors.^25–30^ In this regard, there is an emerging trend to improve the functional properties of PC–PBT blends as well by the addition of carbon-based nanofillers. Huang et al. (2018) investigated the structure and property changes of PC–PBT blends induced by CNT addition via melt-compounding. Reportedly, a certain level of phase separation between PC and PBT components was achieved by CNT addition, resulting in enhanced toughness and strength for the ternary blend composites, with the additional aid of facilitated crystallization of PBT phase adding to the improvement in mechanical properties. The formation of a percolation network after a certain filler ratio and an excellent antistatic performance by the blend nanocomposite with 2 wt % filler was also reported.^16^ In another work, the effect of multiwalled carbon nanotubes (MWCNTs) on the thermal, mechanical, and morphological properties of PBT–PC blends were examined.^31^ Significant improvements in the mechanical properties such as 60% increase in tensile modulus, 80% increase in flexural modulus, and ∼57% increase in ultimate tensile strength with small amounts of MWCNT were reported. The heat distortion temperature (HDT) was also reported to increase from 127 to 205 °C, attesting to an improvement in thermal stability at high temperatures.^31^ Wen and Zheng (2019) examined the effect of selective graphite nanoplate (GNPs) dispersion on the electrical and thermal properties of the PC/PBT mixture. The segregated distribution, especially within a cocontinuous structure, significantly enhanced the thermal and electrical conductivities of the PBT–PC/GNP composites. The addition of 3% vol. GNPs was reported to increase the composite’s electrical conductivity by 8 orders of magnitude and its thermal conductivity by approximately 10%.^32^ Graphene derivatives such as graphene nanoplatelets (GNPs) with large surface areas^33–35^ are in the focus of tribological studies as well^36–42^ since they can improve tribological performance by enhancing the thermal conductivity and reducing friction through enhanced interlayer shearing between multiple GNP layers.^43,44^ Karteri et al. (2023) examined the effect of GNPs on the tribological response of the blends of polypropylene (PP) and acrylonitrile butadiene styrene (ABS), and reported improved wear resistance up to the filler weight ratio of 3% after which an agglomeration-induced nonhomogeneous filler dispersion was reported.^36^ Taromsari et al. (2019) examined the combined effect of GNP and hydroxyapatite (HAp) nanoparticles on the tribological, tensile, and biofunctional response of ultrahigh molecular weight polyethylene (UHMWPE) and reported up to 54% reduction in coefficient of friction and up to 82% reduction in the wear rate.^42^

As indicated by the above brief literature survey, despite the interest in improvement of mechanical, thermal, and electrical properties of PC–PBT blends through various routes, no attempts to assess the tribological response of PC–PBT/GNP blend nanocomposites is made, considering the importance of wear resistance of polymer-based materials in automotive and aviation industries. The present work thus aims to investigate the effect of GNP addition on various properties of PC–PBT blends with a particular focus on their tribological response. For this purpose, the weight ratio of PC:PBT was kept constant as 50:50 based on the synergistic effect reported for this PC:PBT weight ratio.^45^ PC–PBT/GNP nanocomposites with 0.5, 1, 3, 5, and 7 wt %. GNP filler ratios were produced via melt-compounding, and afterward microstructural investigations and mechanical and tribological tests were performed on the neat and filled composite blends. Pin-on-disc test method, preferably used to evaluate two-body sliding wear, was applied as the wear test method and AISI 52100 steel ball was chosen as the sliding counterface material to particularly simulate the polymer–metal contact on molded PC–PBT blends under moderate loads for automotive and aviation applications. The experimental and theoretical results were discussed in relation to each other and the literature reports.

## 2 Materials and Method

### 2.1 Materials

PC (LUPOY 1303EP-22) and PBT (PIMADURE HS40 N) were supplied by Aydin Plastic Co. Ltd., Turkey. Graphene Nanoplatelet-GNP (NG01GNP0109G25) with purity >99.9%, size: 3 nm, S.A: 800 m^2^/g, Dia: 1.5 μm, (25 g) was purchased from Nanografi Nano Technology (Turkey). PC had a melt flow index (MFI) of 22 g/10 min (300 °C/1.2 kg) and a density of 1.2 g/cm^3^. PC has a tensile modulus of up to 2.3 GPa, and a tensile strength of up to 66 MPa.^46^ PBT had an MFI of 30–45 gr/10 min (250 °C/2.16 kg) and a density of 1.31 g/cm^3^. It has a tensile modulus up to 2.4 GPa, and tensile strength up to 60 MPa.^47–49^

### 2.2 Preparation of Samples

Polymer nanocomposite samples were prepared via melt-compounding using a melt mixer (Kökbir RTX-M40-Turkey). During the melt mixing process, 260 °C was set as the melt temperature, and the screw speed remained at 30 rpm. The resulting material was shredded and further processed in a filament manufacturing system (Filamex Fx-20-Turkey) equipped with a 20 mm diameter single screw extruder and 2–3 mm nozzles. Afterward, the filaments were put into granule form using a granulator (KÖKBİR-Turkey). Film samples for characterization purposes and plate samples with required standard dimensions for mechanical tests were produced from the prepared nanocomposite granules via compression molding under 250 °C melt temperature and 5 MPa pressure using a Kökbir brand and RTX-Px model (Turkey) compression molding system with 30-ton capacity, equipped with heating and cooling tables. Prior to the compression molding process, nanocomposite samples in granule form were kept in an air-blown oven at 100 °C for a period of 6 h. PC/PBT weight ratio was maintained as 1:1 for all nanocomposites whereas the filler ratio within the composites varied as 0.5, 1, 3, 5, and 7 wt %. 1:1 PC/PBT ratio was chosen based on the favorable physical property improvements reported at this blend ratio ascribed to a cocontinuous phase structure.^32^ Weight fractions of the nanofillers were determined based on a range of studies reporting optimum filler fractions providing the highest mechanical, thermal, and electrical attributes for thermoplastic blends reinforced with carbon-based nanofillers.^36,50–53^ In sample notation “G-x”, G denotes the GNP content, and *x* denotes the weight percentage of the filler. PC–PBT blend without filler content is simply referred to as the neat or “PC–PBT” sample. The flow diagram of the sample preparation process is presented in Figure 1.

**Figure 1:**
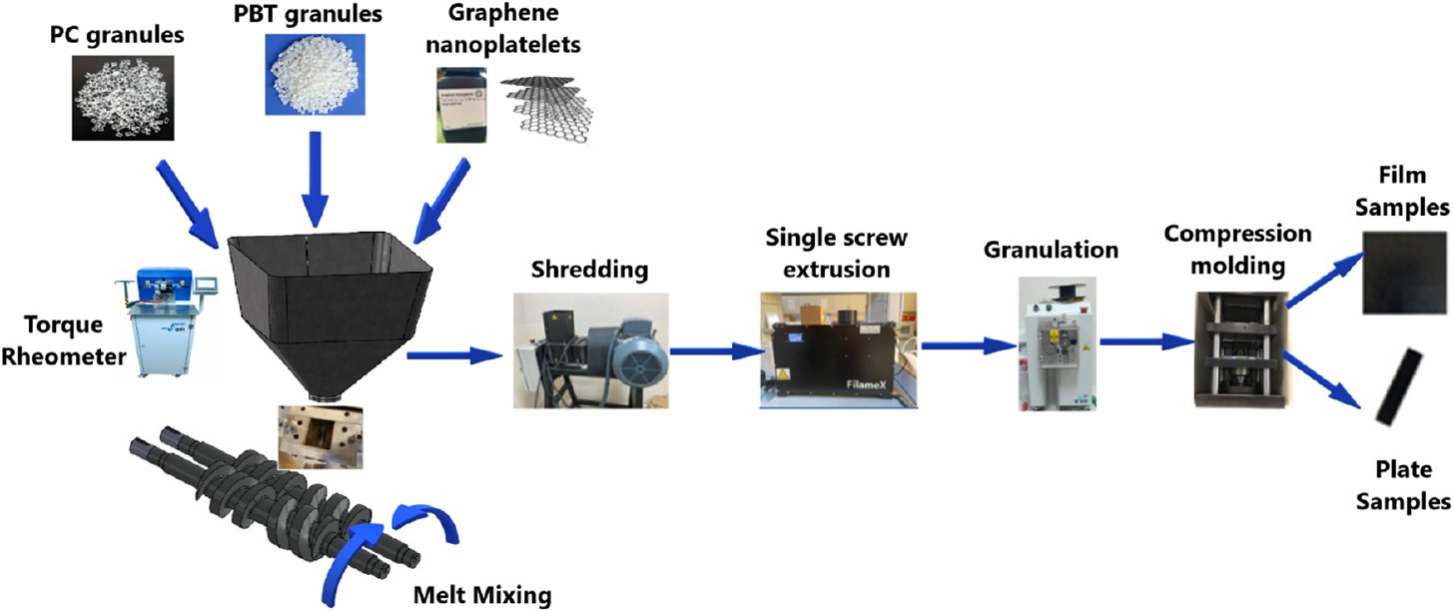
Flow diagram of the sample preparation process.

### 2.3 Scanning Electron Microscopy (SEM)

For the characterization of phase morphologies of the neat blend and nanocomposite samples, a Tescan (MAIA3 XMU – Czech Republic) brand scanning electron microscope was used at an operating voltage of 5 kV. Prior to the SEM analyses, the samples in thin sheet form were cold-mounted vertically for ease of observation under SEM. After cold mounting, the free ends of the film samples corresponding to each filler ratio were subjected to cryogenic fracturing in liquid nitrogen, and the revealed fractured cross-sections were coated with a thin gold layer to enable conductivity prior to SEM analyses.

### 2.4 Measurement of Mechanical Properties

Tensile, three-point flexural, and un-notched Izod impact tests were carried out at room temperature. Tensile properties of the test samples prepared in thin sheet form were measured according to ASTM D882 using an AHP brand (Turkey) universal tensile test rig equipped with a 5kN load cell to increase sensitivity. The tests were carried out with a constant cross-head speed of 10 mm/min. Un-notched Izod impact tests were performed on an AHP brand (Turkey) universal (Izod-Charpy) impact tester equipped with a 5 J pendulum, according to ASTM D4812 using the test samples prepared in dimensions of 10 × 70 × 3 mm. The flexural strength of the produced samples was evaluated with three-point flexural tests on AHP brand (Turkey) universal testing equipment with a 20 kN capacity according to ASTM D790. Shore-D hardness measurements were performed using a hand-held analogue durometer. For reproducibility of the test data, each test was performed 3 times, and the average values are reported.

### 2.5 Tribological Tests

Prior to the tribological tests, all sample surfaces were ground to obtain a surface roughness of Ra ≈ 0.02 μm and subjected to ultrasonic cleaning by submerging in acetone. Wear tests were performed at room temperature with the rotating mechanism of a hybrid Turkyus Brand pin on a disc test rig (Turkey) that can perform both rotating and reciprocating wear tests. Sliding wear tests were performed under 5 and 10 N loads at a sliding speed of 0.115 m/s. AISI 5200 steel (0.93–1.05% C, 0.15–0.35% Si, 0.25–0.45% Mn, 1.35–1.60% Cr) was used as the sliding counterface material. For reproducibility of specific wear rate data, each test was performed three times, and the average values were used in the calculation of specific wear rates. A single selected data set was used to obtain coefficient of friction (COF) graphs. Frictional forces received from the load cell mounted on the moment arm of the test rig were instantaneously recorded in a log file, which was then used to plot the COF vs time curves under respective loads. Average COF values for each sample were calculated by taking the average of 600 COF data received over the sliding period during each wear test. Wear test parameters are listed in Table 1. The frictional forces recorded in a log file were used to obtain the coefficient of friction (COF) data. During the wear tests, flash temperatures at the contact zone of the tribo pairs were captured using a Bosch GTC 400 °C (Germany) thermal camera. Temperature measurements were performed five times within the same interval for each test, and the average values were reported. A thermal camera image showing the contact temperature on a sample rotating in relative motion to the steel ball is shown in Figure 2. Wear profiles for each test were obtained with a Filmetrics Profilm3D optical profilometer and the worn volume data were obtained by multiplying the average of 15 cross-sectional wear track areas (3 wear tracks × 5 measurements) by the diameter of the wear track. Worn volume data were then used to obtain the specific wear rate data by normalizing the worn volume with the applied normal load and the sliding distance as per the following eq 1:

1 $$w=\frac{V}{\mathrm{FL}}m^{3}N^{-1}m^{-1}$$

where; V denotes the average worn volume (m^3^), F is the normal load (N), and L is the total sliding distance that the abrasive ball traveled during the test.

**Figure 2:**
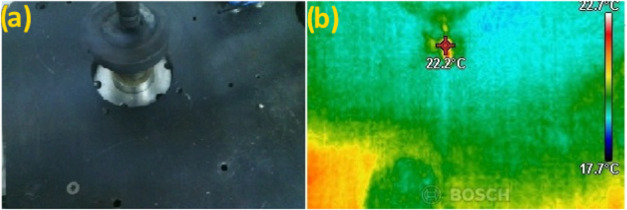
(a) Image of the test rig and (b) thermal camera image of the sample surface.

**Table 1:** Dry Sliding Wear Test Parameters

steel ball hardness	62 Rockwell C
steel ball diameter	6 mm
loads applied during the wear tests	5 Newton, 10 Newton
rotational speed of sample holder	220 rpm
track radius	5 mm
sliding speed	0.115 m/s
sliding distance	138 m
data flow rate	0.5 Hz
ambient humidity	44%
ambient temperature	room temperature

The maximum contact pressures for the applied loading conditions were calculated in accordance with the following eqs 2–5.^36,54,55^

2 $$\frac{1}{E^{*}}=\frac{\left(1-v_{1}^{2}\right)}{E_{1}}+\frac{\left(1-v_{2}^{2}\right)}{E_{2}}$$

3 $$a^{3}=\frac{3\mathrm{FR}}{4E^{*}}$$

4 $$\frac{1}{R}=\frac{1}{R_{1}}+\frac{1}{R_{2}}$$

5 $$P_{\max}=\frac{3F}{2\pi a^{2}}$$

where E* is the reduced Young’s modulus, *E*_1_ and *E*_2_ are Young’s moduli of the tribo pair, *v*_1_ and *v*_2_ are the Poisson’s ratios of the tribo pair, a is the radius of contact area, F is the applied load, *P*_max_ is the maximum contact pressure, and *R*_1_ and *R*_2_ are the radii of contact geometries.

## 3 Results and Discussion

### 3.1 Microstructural Changes

Figure 3 shows the cryo-fractured cross-sectional surface morphologies of the samples with 0.5, 1, 3, 5, and 7% filler weight ratios. The miscibility indicated by the absence of macro-multiphase features in Figure 3 can be ascribed to the transesterification reaction between the blend constituents that takes place during the melt-compounding process.^16^ Transesterification reaction takes place at the interface of PC and PBT phases, and most of the time, it is inevitable. Among PC and PBT, transesterification reaction has a higher impact on the semicrystalline PBT phase, which has certain implications on the mechanical properties of the overall blend or composite. As the weight or volume ratio of the PBT in the blend is reduced, leading to smaller PBT phases, the crystallinity is also impaired resulting in a higher transesterification degree.^56^ In the present case, the weight ratio of 50:50, applied for all samples, resulted in a limited transesterification degree, yet a certain degree of miscibility between the phases seems to be maintained. In the SEM images of the nanocomposite samples (Figure 3) one can see two distinct tones, i.e., lighter and darker regions on the fractured surfaces of the cross-sections. Although a macro-multiphase feature is absent on all samples, phase separation to a certain degree is evident on the SEM images. In another report on the effect of carbon nanotubes (CNTs) on the microstructure and property changes of PC–PBT blends, Huang et al. (2018) reported that in miscible blends CNTs selectively tend to adsorb the molecular chains of the blend constituent which has the higher interfacial reaction affinity among the two materials, leading to a phase separation between the two constituents.^16,57,58^ In the present case, the same phenomenon is believed to apply for GNPs, which is another carbon-based material and basically the unfolded version of multiwalled carbon nanotubes (MWCNT), as some local regions in Figure 3 are decorated with bubble-like light tones. These tones represent the blend constituents of the nanocomposite which has higher interfacial affinity, i.e., with a higher number of molecular chains for GNPs to adsorb.

**Figure 3:**
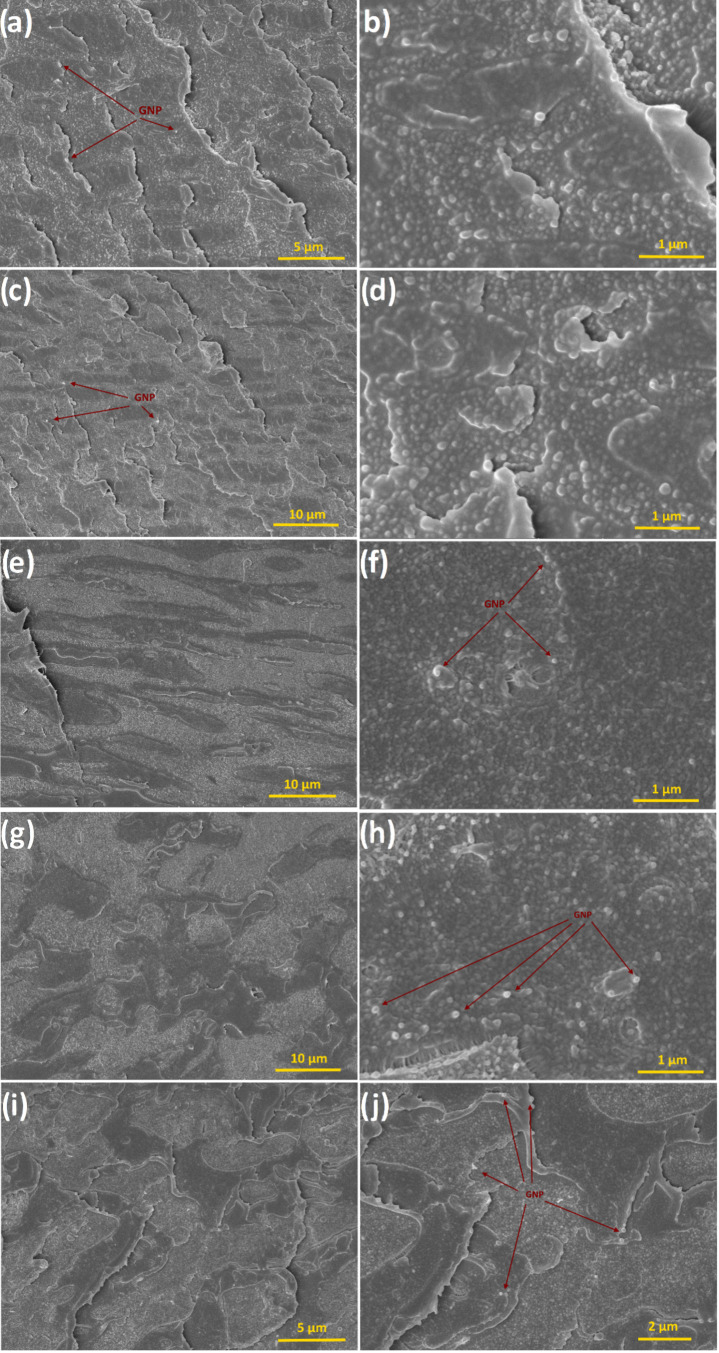
(a, c, e, g, i) Low- and (b, d, f, h, j) high-magnification SEM images of Cryo-fractured cross-sections of G-0.5, G-1, G-3, G-5, and G-7 film samples, respectively.

In Figure 3 the separation between the light regions (the phase on which GNPs selectively adsorb) and the dark regions (the phase that is subject to a lower degree of interfacial bonding with GNP) gradually increases with increasing filler ratio up to the filler fraction of 7 wt % GNP (Figure 3i,j) leading to a higher extent of phase separation between the blend constituents. In Figure 3a–d, representing the fractured surface of G-0.5 and G-1 samples, almost all cross-section surfaces are decorated with bubble-like light regions, whereas in Figure 3e,f (G-3 sample’s cross-section) the dark regions become more distinct. This distinction is gradually increased in both vertical and horizontal directions in Figure 3g,h representing the sample with 5 wt % GNP. At 7 wt % GNP filler fraction, the extent of bright areas is reduced (Figure 3i,j). This phenomenon can be ascribed to agglomeration and saturation effects at increased filler ratios. At low-to-moderate filler ratios, polymer chains selectively adsorb on polymer chains leading to distinctly phase-separated morphologies as in the cases of 0.5, 1, 3, and 5 wt % GNP ratios (Figure 3a–h), whereas at higher concentrations, GNPs are inclined to form aggregates as a result of strong van der Waals forces arising from the high aspect ratio of fillers,^52^ and this leads to restricted surface area for interaction of GNPs.

On all figures, the protrusions indicated by arrows are GNP particles loosely attached on the molecular chains of the respective blend constituent. The phase separation between the constituents of the PC–PBT blend and the factors affecting its extent have been studied and reported by several researchers. As reported by Wilkinson and Tatum (1996), the structural development of the blends of PBT is mainly controlled by the rapid crystallization behavior of PBT initiating below its *Tm* (220 °C), even though under its spinodal temperature (*Ts*) of 198 °C, PC–PBT blend (50:50) is expected to demonstrate phase mixing, hence a homogeneous mixture.^59^ The authors relate this behavior to the rapid crystallization kinetics of PBT that supersedes the lower critical solution temperature (LCST) type phase behavior of PC–PBT blend (50:50), resulting in a slow phase dissolution progress.^59^ Although crystallization of PBT is restricted by the transesterification reaction,^60^ it is reported to be improved by the addition of carbon-based fillers such as CNT into the blend since this leads to a higher crystallization temperature (*T*_c_).^16^ This also explains the higher extent of phase separation with an increasing filler ratio of GNP (Figure 3). Apart from the crystallization kinetics of PBT leading to phase separation in binary PC–PBT blends, increasing the filler ratio in ternary blends of PC–PBT is believed to induce a phase separation through increasing degree of agglomeration as well. As reported for CNTs^16^ and GNPs^36^ increasing the filler ratio results in a less homogeneous dispersion of the filler within the nanocomposite structure, hence impaired mechanical properties which is intrinsic to most composites prepared by melt-compounding either with a melt mixer or a single or twin-screw extruder under shear stress. Noorunnisa (2016) reported improved moduli values up the filler ratio of 4 wt % for ABS/GNP composites and Karteri et al. (2023) reported such improvement up to the filler ratio of 3 wt % for PP-ABS/GNP composites.^36,61^ Authors ascribed reduced dispersion and consequent reduction in mechanical properties after this filler ratio to GNPs’ strong intermolecular bonding which cannot be broken with shear stress after a certain filler ratio, in addition to the weak bonding between GNPs and matrix phases.^36^ As elaborated above, several mechanisms are decisive on the composite structures in the particular case of nanocomposites of PC–PBT blends prepared through melt-compounding, and these mechanisms are interrelated with each other.

### 3.2 Mechanical Properties

Figure 4 shows the stress–strain curves obtained from the tensile and 3-point bending tests of the produced samples. Tables 2 and 3 provide the modulus, strength, and elongation data acquired from the respective tests. It can be observed from Figure 4 that the tensile modulus and elongation at break of the samples gradually increase with increasing filler ratio up to the fraction of 5 wt % after which they show a slight decline. In amorphous-semicrystalline blends such as PC–PBT, the presence of PBT crystallites is largely held accountable for tensile ductility as the slip and distortion of lamellae across the stress direction can absorb a considerable amount of energy.^16^ The same behavior applies for the 3-point flexural test results as well. This is indicative of a strong interaction between the filler and the polymer matrix. The weak van der Waals interaction between the matrix and the filler suffices to transfer the stress at small strains, while the tensile and flexural ductility of the material governs the deformation behavior at higher stresses, resulting in break at higher elongation and higher ultimate strengths. Reportedly, the aggregation state of GNPs increases with increasing volume ratio of GNPs, which obstructs the continuous improvement of mechanical properties of samples, and beyond a certain threshold, deterioration of mechanical properties starts.^62^ Thus, aggregation or agglomeration of GNPs with increasing filler ratio, deterioration of crystallinity, and the resulting tensile brittleness are held responsible for the decline of tensile and flexural properties with increasing filler ratio after 5 wt % filler fraction. Moreover, melt-mixing-induced modifications on the morphology of GNPs have been reported to reduce their load transferring and lubricating capabilities by altering their aspect ratio,^63^ which is more likely at higher filler ratios under the thermomechanical conditions of melt-compounding.^64^ In Figure 4, the slight decline in tensile-flexural strength and elongation at break can be ascribed to a higher extent of agglomeration at a 7 wt % filler ratio leading to discontinuity in mechanical properties. The adverse effect of nanofiller agglomeration on the crystallinity of PC/PBT blends is also reported by Oge (2025) who reported a reduction in the calculated crystallinity of PC/PBT/GNP nanocomposites from *X*_c_ = 44.40% at 3 wt % GNP ratio to *X*_c_ = 30.57 at 7 wt % GNP ratio by using differential scanning calorimetry (DSC) data.^52^ This also applies for the flexural strength, which exhibited a decline after the filler ratio of 5 wt %.

**Figure 4:**
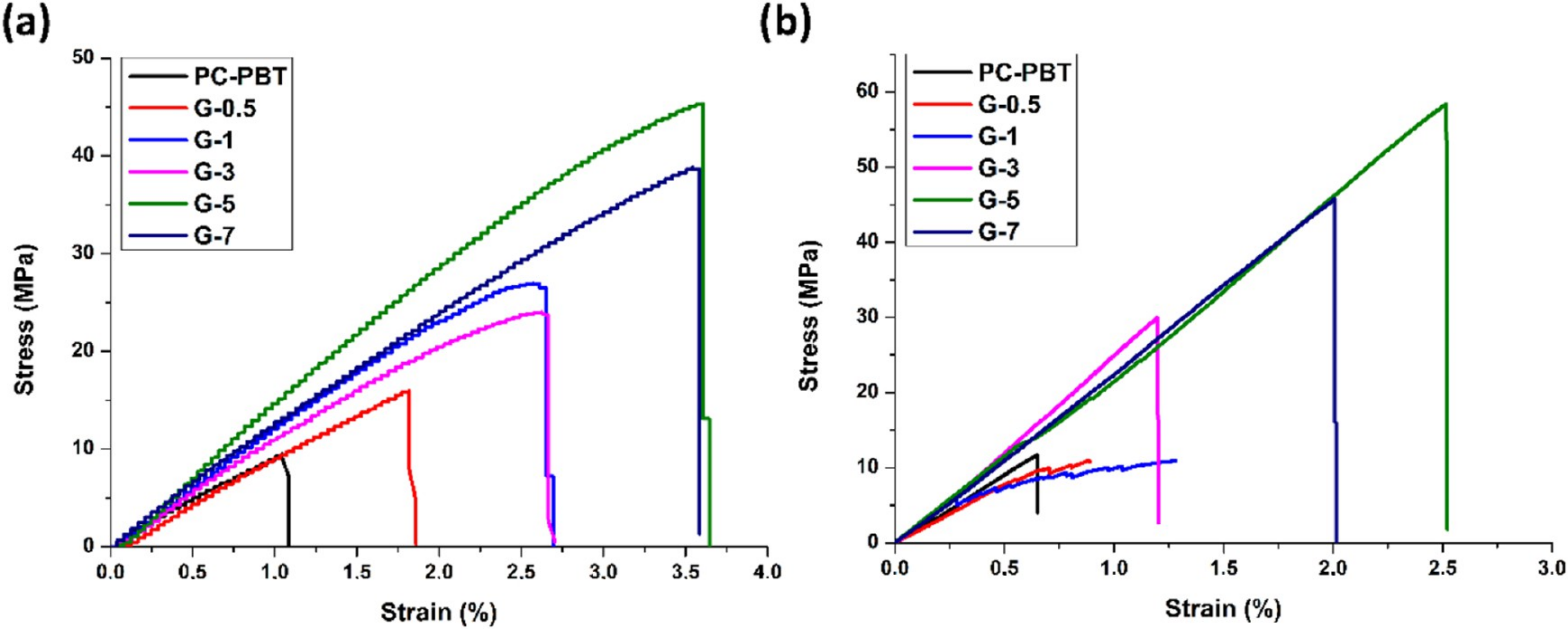
Stress–strain curves obtained from (a) tensile and (b) three-point bending tests of the produced samples.

**Table 2:** Tensile Test Results^a^

**samples**	***E**_**t**_*	***T***_**s**_	***e***_**B**_
	**MPa (modulus)**	**MPa (tensile strength)**	**(ductility)**
PC/PBT	976.43 ± 46.98	9.52 ± 1.88	1.09 ± 0.11
G-0.5	1009.5 ± 50.96	15.96 ± 2.52	1.86 ± 0.14
G-1	1214.3 ± 64.96	26.97 ± 3.90	2.69 ± 0.28
G-3	1235.4 ± 67.16	24.03 ± 3.59	2.7 ± 0.26
G-5	1429.7 ± 68.4	45.37 ± 4.88	3.65 ± 0.37
G-7	1333.6 ± 61.3	38.92 ± 3.70	3.59 ± 0.33

a Each value represents the average of 5 test results.

**Table 3:** Three-Point Flexural Test Results^a^

**sample**	***E***_**f**_ **(MPa)**	***F***_**s**_ **(MPa)**	***e***_**B**_ **(%)**
PC/PBT	1683.5 ± 101,22	11.67 ± 1.28	0.65 ± 0,08
G-0.5	1800.5 ± 93.22	10.95 ± 1.33	0.88 ± 0.11
G-1	1952.5 ± 105.43	10.97 ± 1.42	1.22 ± 0.16
G-3	2241.5 ± 108.44	29.96 ± 3.25	1.27 ± 0.15
G-5	2329.9 ± 106.53	58.42 ± 4.67	2.52 ± 0.19
G-7	1943.6 ± 99.58	45.73 ± 4.13	2.01 ± 0.17

a Each value represents the average of 5 test results.

Figure 5 shows the un-notched Izod impact test results for the samples with varying filler ratios. As indicated by the values, the impact strength value is consistent with the elongation at break for both tensile and 3-point bending tests. The consistency in the samples’ tensile, flexural, and impact behavior can be observed from Tables 2, 3 and Figure 5. The samples’ flexural toughness resembles its tensile behavior except for the slight increase in toughness in the case of G-3. The lowest impact strength (Figure 5) is observed with the neat sample, whereas G-5 exhibits the highest impact strength which is also ∼49% higher as compared to the neat blend (3361 for G-5 vs 1833.1 for PC–PBT). Obviously, the sample with 5 wt % filler ratio succeeds to withstand dynamic load in the form of sudden impact and absorbs more energy, while also enduring slowly applied static loads as in the cases of tensile and flexural tests. The deterioration of toughness after the optimum filler ratio of 5 wt % can be attributed to the reduced mobility of macromolecules induced by the particles resulting in the development of a discontinuous interphase around the particles, hence a slight reduction in elongation values occurs (Table 3) due to the discontinuous mechanical properties.

**Figure 5:**
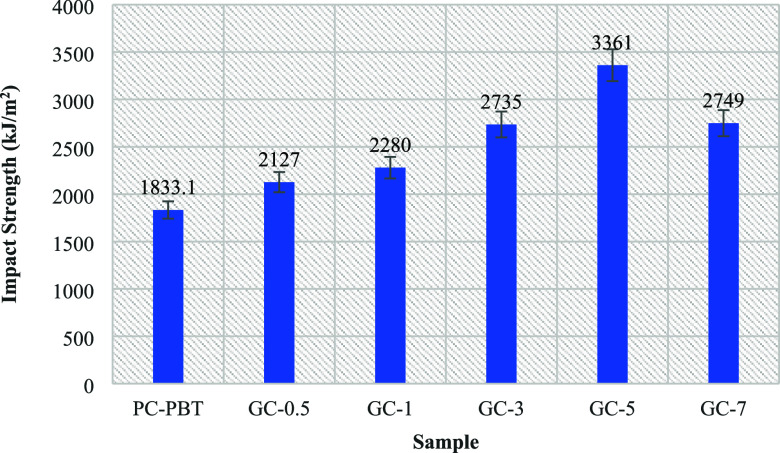
Un-notched Izod Impact test results of the neat PC–PBT blend and nanocomposite samples with varying filler ratios (each value represents the average of 5 test results).

### 3.3 Tribological Results

#### 3.3.1 Friction and Wear Data

During the wear process, the amount of worn volume is directly affected by the applied normal load and the resultant contact pressure which can be theoretically calculated by the physical properties of the tribo pair.^36^ Accordingly, the maximum Hertzian static contact pressures, maximum shear stresses, depths of maximum shear stresses, and circular contact area diameters were calculated in accordance with the tensile elastic moduli of each sample received from the tensile tests (Table 2) and shown in Table 4 (elastic modulus of the steel ball: 210 GPa; the diameter of steel ball and flat surface: 6 mm and ∞, respectively; Poisson’s ratio of steel ball and polymer surface: 0.3 and 0.35, respectively). As shown in Table 4, the tensile modulus of elasticity increases with increasing GNP content up to the optimum filler ratio of 5 wt % after which it shows a slight decline. Maximum contact pressure (*P*_max_) and maximum shear stress (τ_max_) calculated from the moduli follow the same trend, whereas the geometrical parameters (the depth under which max shear stress occurs (z) and contact area diameter (2a)) decline gradually with increasing filler ratio. Such a decline in the theoretical depth (z) and width (2a) of penetration is the result of increasing stiffness (*E_t_*) which is expected to act against the penetration of the steel ball into the worn surface.

**Table 4:** Max. Contact Pressure, Max. Shear Stress, Depth of Max. Shear Stress, and Circular Contact Area Diameters Calculated for Each Tribo Pair

	*E_t_* (GPa)	load (N)	*P*_max_ (Mpa)	τ_max_ (Mpa)	*z* (mm)	2a (mm)
PC–PBT	0.976	5	50.9	15.2	0.108	0.433
		10	64.1	19.1	0.136	0.546
G-0.5	1.009	5	52	15.5	0.107	0.428
		10	65.5	19.6	0.134	0.540
G-1	1.214	5	58.8	17.6	0.1	0.403
		10	74.1	22.1	0.126	0.508
G-3	1.235	5	59.5	17.8	0.1	0.401
		10	74.9	22.4	0.126	0.505
G-5	1.429	5	65.5	19.6	0.095	0.382
		10	82.5	24.7	0.12	0.481
G-7	1.333	5	62.6	18.7	0.097	0.391
		10	78.8	23.5	0.122	0.492

Coefficient of friction (COF) and specific wear rate data are two important experimental indicators for the tribological behavior of tribo pairs that are in contact under various conditions. Coefficients of friction data are derived from the frictional forces instantaneously measured during the tribological tests, whereas specific wear rates are theoretically calculated from the cross-sectional area of worn tracks multiplied by the track diameters, which are then normalized with sliding distance and normal load to eliminate the direct effect of these parameters on the worn volume to provide a clearer insight into the effect of other parameters such as the filler ratio. Accordingly, the COF and specific wear rates may not be necessarily correlated but provide valuable information from different aspects. Figure 6 shows the variation of COF data against sliding time, whereas Figure 7 shows the average COFs calculated over the entire sliding time. As indicated by the average COF values in Figure 7, coefficients of friction under 5 N are significantly higher than those under 10 N for all filler ratios. The neat PC–PBT blend exhibited the highest average COF values under 5 and 10 N at 0.47 and 0.31, respectively, whereas the overall lowest COF was exhibited by G-5 with 0.25 (with 24% reduction under 10 N) and 0.37 (with 27% reduction under 5 N). The reduction in COF under both loading conditions can be ascribed to the lubricating effect stemming from the enhanced interlayer shearing between GNP layers which is also reported by other tribology studies on use of GNPs.^43,44,63^ Moreover, as indicated by the thermal camera measurement results shown in Figure 8, the neat PC–PBT blend exhibits relatively higher contact temperatures under both loads, whereas slight deviation is observed among the filled nanocomposites. This is attributable to the graphene constituent’s acting in favor of heat dissipation and against matrix degradation due to its high thermal conductivity^43,63,65^ which seems to provide a conducting network as from the lowest filler ratio, thus contributing to the better COF performance of the filled nanocomposites. The explanation for the slight increase of COF in the subsequent filler ratio (G-7) would be the higher aptitude of GNPs to form agglomerations on the sliding surface at high concentrations, resulting in discontinuity of mechanical contact between the tribo pairs. The reduced COF values with increasing load is attributable to formation of a more stable tribofilm acting as a barrier against direct contact between the tribo pair.^44^

**Figure 6:**
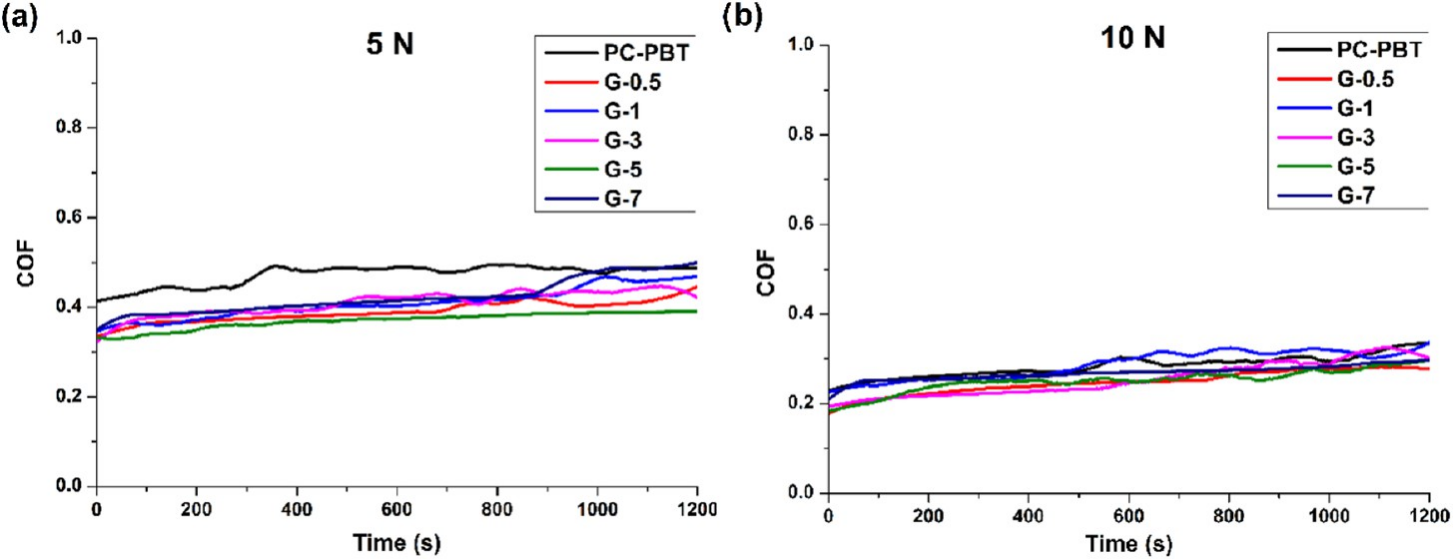
Coefficient of friction (COF) graphs of samples with varying filler ratio under (a) 5 N load and (b) 10 N load.

**Figure 7:**
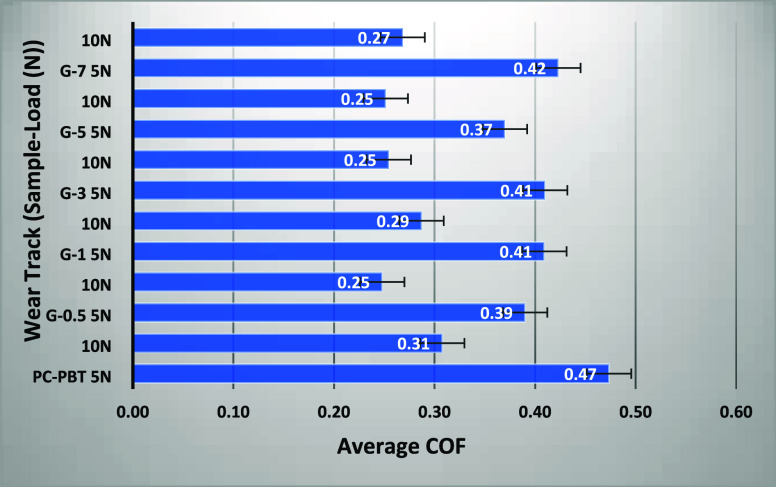
Average coefficient of friction (COF) data for samples with varying filler ratios worn under varying loads (each value is the average of 600 data points (for 0.5 Hz data flow rate) from the respective COF curve data in Figure 6).

**Figure 8:**
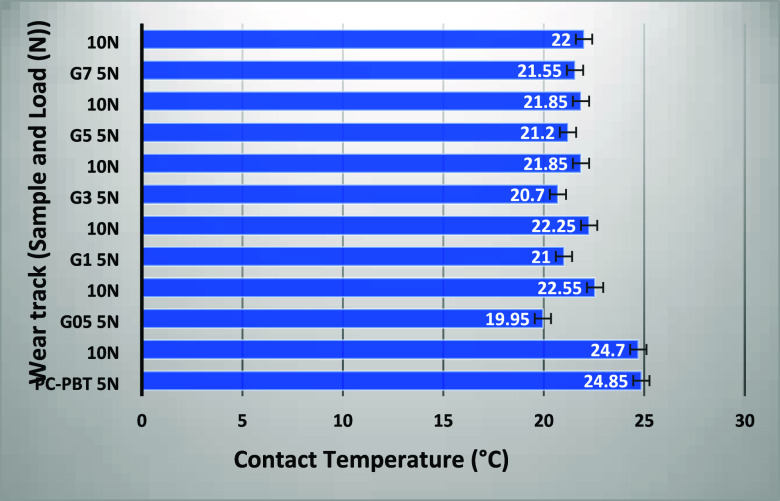
Contact temperatures captured from contact surfaces during the dry sliding wear tests (each value represents the average of 5 measurement results).

The distinction between the wear performance of the neat and the filled specimens is more pronounced on the specific wear rate data (Figure 9) as it implies a much more significant achievement of ∼90% reduction with G-5 under 10 N, and ∼86% reduction with the same filler ratio under 5 N, compared to the neat PC–PBT blend. Considering that specific wear rate is related to volumetric wear loss and that volumetric wear loss can be contemplated in three dimensions; the depth of maximum shear stress (1D) and the real contact area (2D) can be held accountable for these three dimensions. Moreover, the amount of max. shear stress can be deemed as the fourth dimension or driving factor for volumetric material removal from the system. Each increase or reduction in the physical properties, (load, material properties, etc.) that affect the calculation of the contact area, the depth, and the amount of shear stress acts as a multiplier in theoretical calculations of wear loss. In Tables 2 and 4, the tensile moduli of the samples gradually increase with increasing filler ratio, thus adding to contact pressure and shear stress while reducing the depth of max. shear stress and the contact area diameter, which are the mentioned theoretical multipliers for volumetric wear loss. Consequently, a gradual reduction in the specific wear rate data would be expected with increasing filler ratio due to the reduction in the theoretical multipliers (*z* and 2a) for wear loss. However, the specific wear rate data (Figure 9) show that a slight deviation is observed among the GNP-filled samples while they all significantly outperform the neat PC–PBT blend. The significant difference between the wear losses of the neat and filled samples can also be clearly observed from the wear track profiles shown in Figure 10 for both loading conditions. As a confirmation of the specific wear rate data, the wear profiles for the filled composites show slight deviations with significantly lower track depths and widths as compared to the neat PC–PBT blend (Figure 10). It can be stated based on these findings; factors other than the mechanical contact parameters stemming from the increasing stiffness of the material surface have also been effective in determining the wear behavior. One of the factors acting against the expected gradual reduction in specific wear rate with increasing filler ratio is the adverse effect of increased nanofiller amount on growth of PBT crystallites through agglomeration in addition to the reduced distance between GNPs, adversely affecting the nucleation mechanism during polymer chain growth.^66,67^ The relatively insignificant increase in the Shore-D hardness results (Figure 11) is also effective against the expected gradual improvement in the wear performance. The factors acting in favor of enhancement in wear performance is the contributive effect of GNP addition through the facilitated formation of a solid tribofilm on contact surfaces reinforced with GNPs, which can prevent the direct contact of the sliding counterface with the material surface as also reported in other studies.^44,65,68^ The contribution of the tribo-film formation and transfer-film mechanism will be further elaborated during the discussion of the tribo-pair analyses. Other contributive factors are the high thermal conductivity of GNPs contributing to the removal of frictional heat from the surface; and the increased resistance of material surface by increasing filler ratio against penetration of abrading ball through increased stiffness and shore-D hardness. Thus, the change in the specific wear rates is subject to a competition between the abovementioned limiting and contributing effects of GNP addition, and it can be stated considering the above discussion supported by the hardness and temperature measurements that the change in specific wear rates with varying filler ratios is related to the presence of GNPs to a higher extent than its weight ratio within the sample composition.

**Figure 9:**
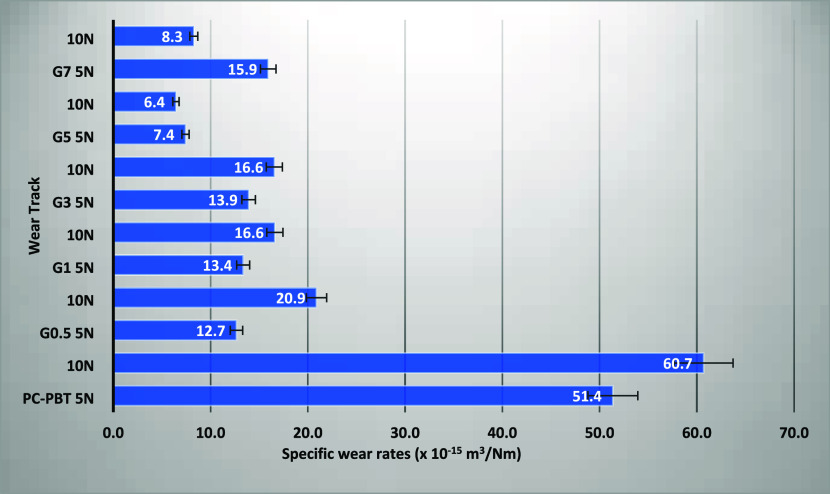
Specific wear rates of samples under 5 and 10 N loads (each value is calculated using 15 measurements from 3 wear tracks).

**Figure 10:**
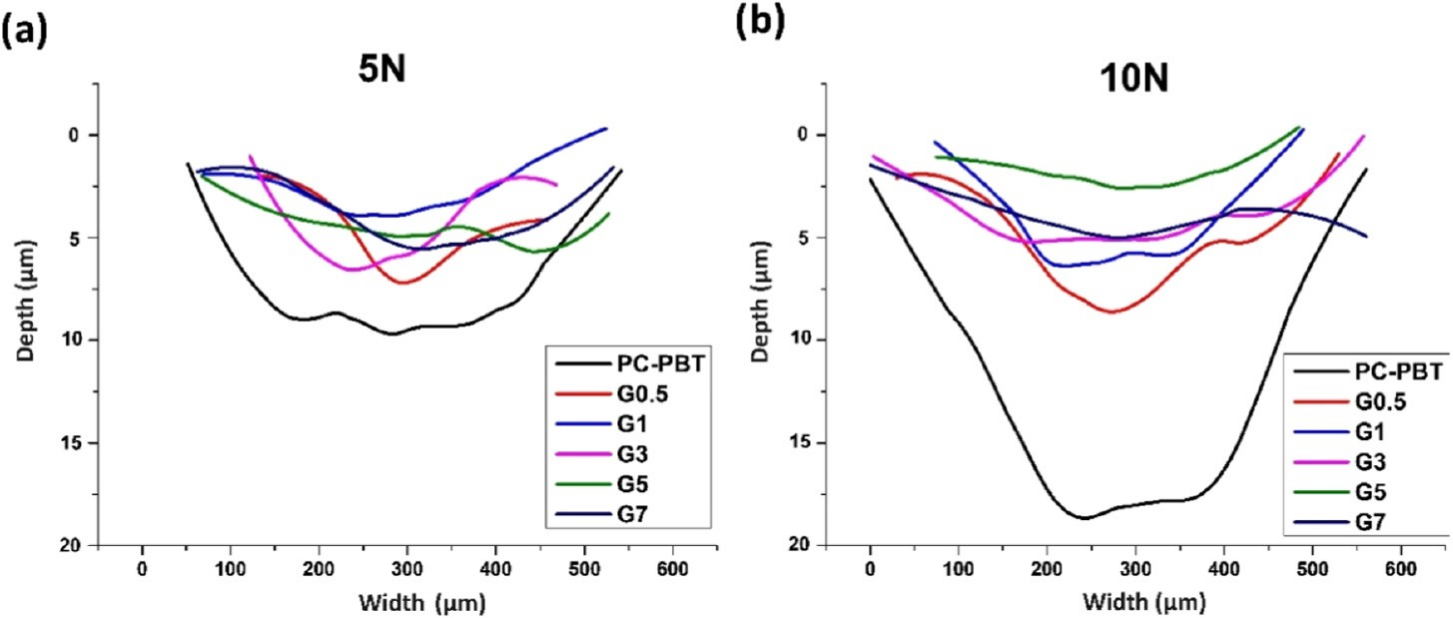
Cross-sectional wear profiles of samples (a) under 5 N and (b) under 10 N loads obtained from 3D profilometer measurements.

**Figure 11:**
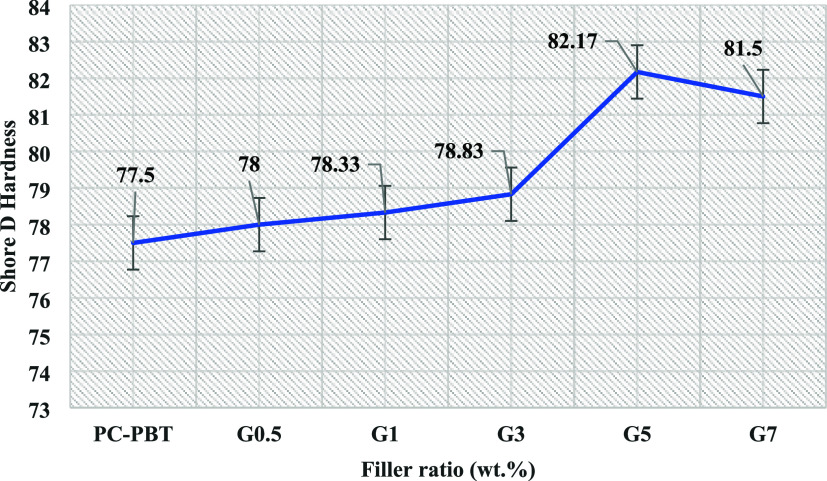
Shore-D hardness test results (each value represents the average of 5 measurement results).

As shown in the Shore-D hardness test results in Figure 11, the lowest hardness belongs to the neat sample (77.5), and the highest value is obtained from G-5 (82.5). Hardness is a measure of the material’s resistance against indentation of a harder material.^69^ The results shown in Figure 11 precisely comply with the mechanical test results, and they also agree with the information about the agglomeration state of GNPs within the polymer matrix, as a more homogeneous dispersion of filler may provide higher resistance against penetration. As this is supposed to be correlated with the penetration of the ball surface into the wear track, the wear data (COF and specific wear rate) implies similar results as to the wear performance of different samples. It can be inferred that, the presence of GNPs improved the resistance of the blend against penetration of the counter-body even with G-7, although different mechanisms such as enhanced shearing of multiple GNP layers;^63^ or the presence of aggregates acting as stress inducers^70^ might have taken over for G-7. The relatively sharp increase from G-3 to G-5 and the decline from G-5 to G-7 can be likewise associated with the optimum dispersion state and the subsequent agglomeration-induced decline, respectively.

#### 3.3.2 SEM and Topological Analyses of Worn Sample Surfaces

Figure 12a–d shows the SEM micrographs of the worn surface morphologies of the neat PC–PBT sample under 5 and 10 N loads, respectively. The centers of both wear tracks along the sliding direction are characterized by smoother morphologies as compared to the edges, as the highest contact pressure arises in the real contact area, and it is reduced toward the edges. The morphological transition toward the edges is more gradual under 10 N where the edges are decorated with adhesive wear-induced detachments (or spallings). However, under 10 N, the detached wear debris seems to be smeared on the wear track resulting in a slightly discontinuous dark tone (Figure 12c) whereas the surface worn under 5 N (Figure 12a,b) is characterized by detached wear debris which could not be integrated into the wear track as in the case of 10 N load, due to the insufficient contact pressure. Moreover, a wave-like discontinuous surface morphology is more evident on the surface worn under 5 N (Figure 12a,b). This is also reflected in the initial running-in stages of the COF curves under 5 (Figure 6a) and 10 N loads (Figure 6b). This is because the contact between the tribo pairs is disrupted by detached wear debris under 5 N, hence the oscillating effect on the COF curve (Figure 6a), whereas such a disrupting effect was eliminated by the incorporation of wear debris into the wear track under higher contact pressure. Thus, the varying contact pressure under 5 N due to the oscillating contact resulted in wave-like formations as highlighted in Figure 12b. Such a transition from plowing or abrading action at the center to signs of adhesive wear-induced delamination toward the edges also implies that the real contact area is governed by a higher extent of material removal from the surface which might be induced by a direct contact between the counter-bodies, which is also indicated by the absence of a transfer film. As also implied by the cross-sectional wear profiles of samples shown in Figure 10, the wear track’s depth and width is significantly higher under 10 N load due to the higher max. shear stress, its depth, and the real contact area under the given loading conditions. The higher depth and width of the wear tracks for the PC–PBT blend is also indicated by the 3D profiler images provided as inset micrographs in Figure 12a,c. In these images, the color scale of 10 N load has a wider range and the 3D profiler image of the contact surface under 10 N is characterized by a more consistent morphology and color tones as expected from the nonoscillating mechanical contact between the tribo pairs under higher load. Figure 12e–h shows the SEM and 3D profiler images of the G-0.5 sample surface worn under 5 and 10 N. When compared to the worn surface of the neat PC–PBT blend shown in Figure 12a–d, the width of the wear track is narrower as also indicated by the wear profiles shown in Figure 10. Despite the higher contact pressure and maximum shear stress calculated for G-0.5 (Table 4), the reason for the formation of a shallower and narrower wear track is the formation of a GNP-induced tribo film which captures the wear debris and integrates it into the wear track through sintering under high contact pressure and contact temperature, thus reducing the extent of volumetric wear loss. GNP-induced tribo-film formation is the primary factor for the significant difference between the specific wear rates (Figure 9) and wear profile depths (Figure 10) between the filled and the nonfilled specimens. The resulting tribo film or the agglomerates of sintered wear debris function as lubricants to reduce COF^71^ and specific wear rates, and the extent of this function is increased with increasing load as implied by the COF and specific wear rate results (Figures 6 and 9, respectively). The surface of the G-0.5 sample worn under 10 N (Figure 12g) is characterized by a darker tone with more distinct boundaries with the nonworn area, indicating a more stable tribo-film formation. As a result of the relative discontinuity of the tribo film, under 5 N load (Figure 12f) wave-like morphological formations are observed (highlighted in circles), which is also reflected in the oscillating COF curves shown in Figure 6. Plastic deformations accompanied by crack formations are observed to a higher extent under 5 N (Figure 12f), due to the relative severity of adhesive and abrasive wear induced by less amount of tribo film. In Figure 12g, a macro-scale groove is observed in parallel to the circular wear track, and its location coincides with the center of the real contact area. This might be because the maximum contact pressure at the center suffices to exceed the yield stress to induce plastic deformation on the tribo film, whereas the pressure toward the edges fails to exceed it and remains in the elastic region, hence the formation of the macro-scale groove at the center. The mentioned macro-groove also manifests itself on the 2D curve highlighted with red color representing the wear profile in Figure 10, as well as in the inset 3D profiler image shown in Figure 12g highlighted with dark blue color. The COF curve of the G.0–5 sample under 10 N in Figure 6b starts to oscillate after 600 s, implying that after this test period the max. contact pressure at the center of the steel ball partially exceeded the yield stress of the formed tribo film, resulting in a less stable wear regime, as also implied by the ascending COF curve (Figure 6b). In Figure 12e–h, the absence of detached wear debris on the wear track, as opposed to those observed in the case of the neat sample (Figure 12a–d) signifies less material removal from the surface which can be attributed to a higher amount of transfer (or tribo film) material retained on the wear track, hence lower wear loss. This also attests to a lesser extent of direct contact between the counter-bodies in the case of G-0.5 compared to neat PC–PBT.

**Figure 12:**
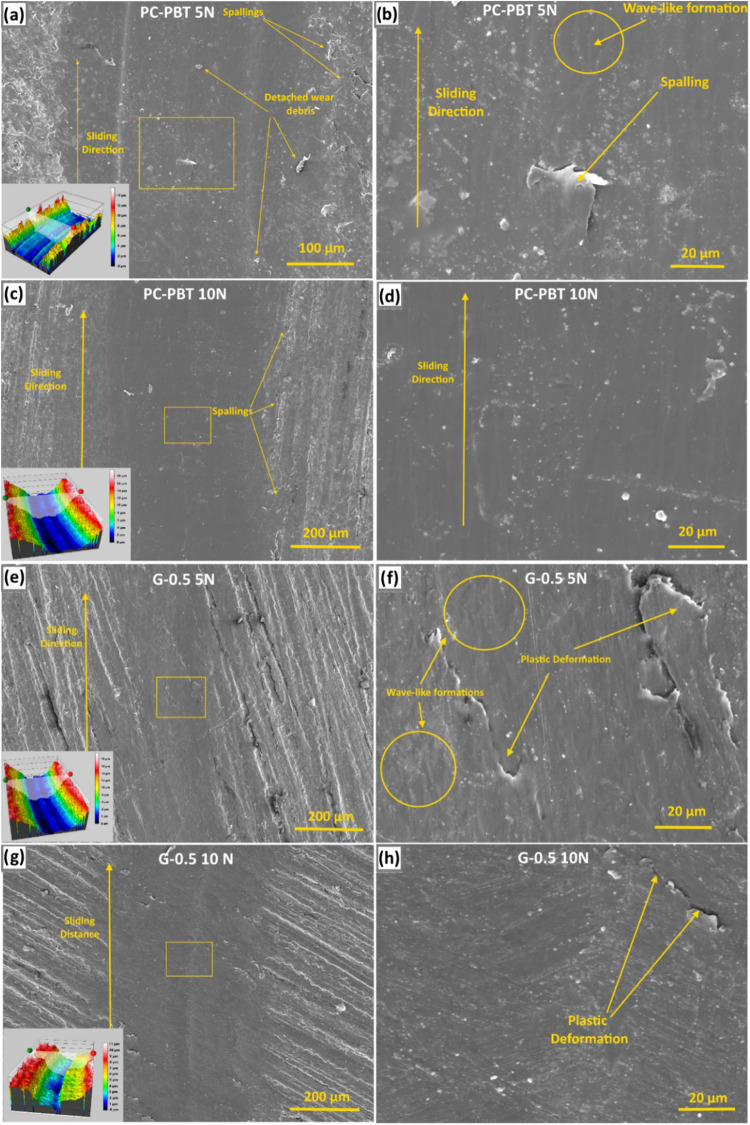
Low and high-magnification SEM and 3D profiler images of the wear tracks for PC–PBT under (a, b) 5 N load and (c, d) 10 N load; and G-0.5 under (e, f) 5 N load and (g, h) 10 N load.

Figure 13a–d shows the SEM micrographs and 3D profiler images of G-1 sample surface worn under 5 and 10 N loads, respectively. The wave-like discontinuities are more apparent under 5 N (Figure 13b), such that they can be even discerned in the macro-scale image (Figure 13a). Such signs of plastic deformation instead of a plowing or abrading action indicate the presence of a transfer film limiting the direct contact between the counter-bodies. The macro-scale groove observed on G-0.5 (Figure 12g) is absent in the case of G-1, however, the wave-like morphology is visible although to a lesser extent than under 5 N. The absence of such a groove under 10 N (Figure 13c) is indicative of whether the yield stress of the tribo film is exceeded across the entire track, or the contact pressure remained under the yield stress throughout the wear track. In Table 4, the tensile modulus (*E_t_*) increased from 1.009 to 1.214 GPa for G-0.5 and G-1, respectively. As another indicator, in Figure 11, the Shore-D harness increases from 78.0 to 78.33 for G-0.5 and G-1, respectively, although insignificantly. The increasing trend of the tensile moduli and the toughness with increasing filler ratio implies that the contact pressure across the contact area of the tribo pairs remains under the yield stress of the forming tribo film, as from the filler ratio of 0.5 wt %, after which a more plastic deformation-oriented regime would be expected due to high hardness and toughness. The wave-like morphologies under 5 N (Figure 13b) are also reflected in the inset 3D profiler images with a noncurvilinear wear profile due to the high modulus encountering a relatively low contact pressure (58.8 MPa) resulting in a nonstable sliding regime. The extent of this behavior is partly reduced with increased contact pressure under 10 N (74.1 MPa) resulting in partial plastic deformation in the form of scaffolding in the sliding direction (Figure 13d). Wedge formations at the edges (Figure 13c) are due to the subsurface fatigue-wear-induced crack formations on the track boundaries where contact of the steel ball with the surface ends, which facilitates the fatigue wear when combined with the surface irregularities.

**Figure 13:**
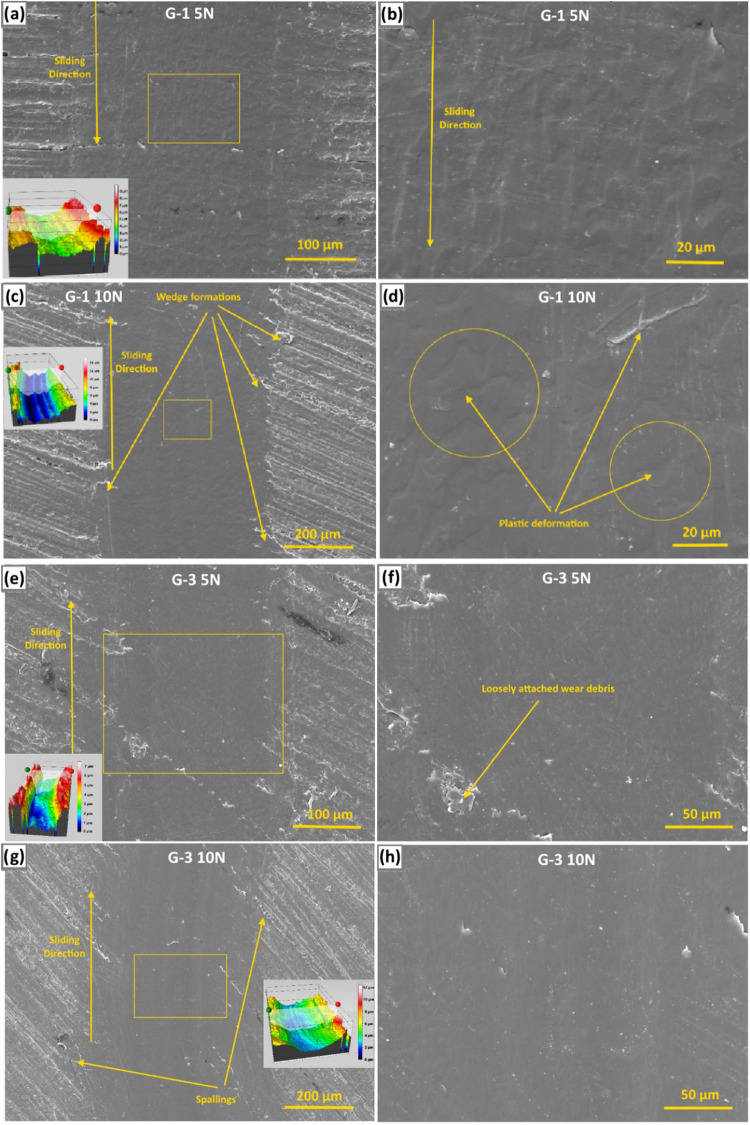
SEM and 3D profiler images of the wear tracks for G-1 under (a, b) 5 N load and (c, d) 10 N load; and G-3 under (e, f) 5 N load and (g, h) 10 N load.

Figure 13e–h shows the surface of sample G-3 worn under 5 and 10 N, respectively. Under 5 N, the continuity of the wear track is disrupted with a macro-scale discontinuity present at the bottom of the inset image indicator rectangle, which can be considered as an extension of the irregularities in the nonworn area, and this can be attributed to the stiff structure of the surface (*E_t_* = 1.235 GPa) encountering a relatively low contact pressure (59.5 MPa) in the presence of wear debris which cannot be entrapped in the tribo layer due to insufficient contact pressure, as also verified by the oscillating COF curve in Figure 6a. Loosely attached wear debris that failed to get incorporated into the tribo film due to insufficient *P*_max_ can also be observed in the detailed SEM micrograph in Figure 13f. Aside from the mentioned irregularities, the dominant wear mechanism seems to be mild polishing wear, as the surface is void of severe abrasive wear signs with a relatively smooth morphology. Besides, in Figure 16h, signs of delamination (with lighter tones) are observed, as a sign of adhesive wear although to a limited extent, which can be induced by a transfer film on the counter-body surface. The overall smoothness of the surface is indicated in the 3D profile image in Figure 16e. The wear track under 10 N (Figure 13g,h) is smoother compared to the 5 N loading condition due to the higher contact pressure (98.2 MPa). This is also reflected in the nonoscillating COF curve until the sliding period of ∼600 s (Figure 6b). The oscillating behavior of the COF curve after this period can be associated with the parallel macro-groove formations which can be discerned in Figure 13g on the sliding surface worn under 10 N as well as the inset 3D profiler image in the same figure. As opposed to the single macro-groove observed at the center of the worn track of G-0.5 in Figure 14c, multiple parallel grooves along the sliding direction are observed across the wear track in Figure 13g, more discernible at the center and diminishing toward the edges. This is also attributable to the aforementioned viscoelastic behavior of polymer-based materials^71^ subject to sliding of a harder counter-material. The difference in the viscoelasticity of G-0.5 and G-3 can be ascribed to the higher stiffness of G-3 (1.235 GPa against 1.009 GPa of G-0.5) due to the higher amount of filler ratio, in addition to the slightly higher Shore-D hardness (78.83) compared to the hardness of G-0.5 (78) as shown in Figure 11, as an indication of higher dispersion of GNPs within the polymer matrix. Hence, there is a more indistinct transition between the plastic and elastic regions across the wear track in the case of G-3, resulting in multiple macro-groove formations because of the interaction between the tribo pair. The dominating wear mechanism is also mild polishing wear under 10 N coupled with signs of transfer-film-induced adhesive wear, except it is milder than under 5 N as also indicated by the more curvilinear wear profile in the 3D profiler image in Figure 13g and the 2D wear profile in Figure 10b.

**Figure 14:**
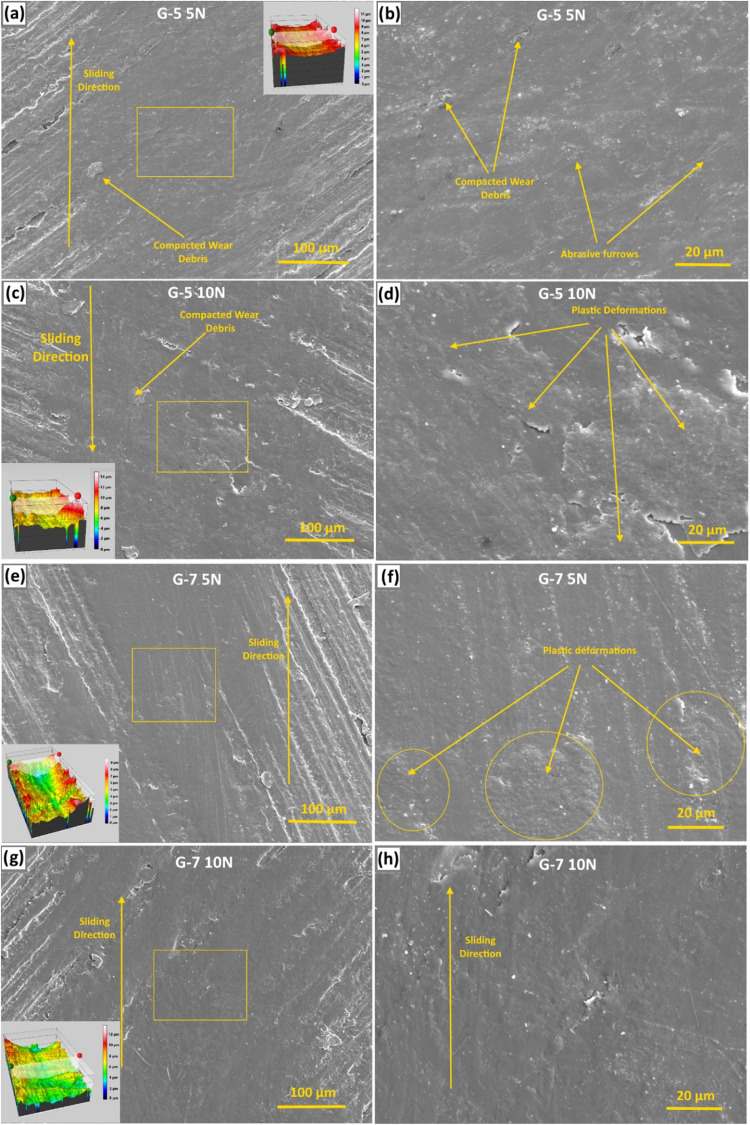
SEM and 3D profiler images of the wear tracks for G-5 under (a, b) 5 N load and (c, d) 10 N load; and G-7 under (e, f) 5 N load and (g, h) 10 N load.

Figure 14a–d shows the contact surfaces of the G-5 sample worn under 5 and 10 N, respectively. As opposed to the relatively smoother surfaces of the samples with lower GNP weight ratios, the wear track of G-5 is characterized by a lumpy morphology which can be attributed to a higher extent of tribo-film formation induced by the higher GNP weight ratio. In recent research by Kumar et al. (2022) on the effect of GNP addition on the tribological performance of glass-fiber reinforced polymer (GFRP) composites, the authors stated that finer wear debris formation on the surface at a specific GNP weight ratio resulted in the formation of a more stable tribo-film layer at the tribo-pair interface, thus reducing the extent of wear loss in GNP-reinforced GFRP composites.^43^ They also attributed the absence of such a stable tribo layer at the contact region of the neat sample to formation of nominal quantity of finer wear debris due to the absence of GNPs^43^ that act as nucleating agents.^72^ Likewise, in the present research, the lowest specific wear rates are achieved by G-5 sample as 7.4 × 10^–15^, and 6.4 × 10^–15^ m^3^(Nm)^−1^ under 5 and 10 N, respectively, which are ∼86 and ∼90% lower than those obtained with the neat blend. Moreover, the lowest average COFs were obtained with G-5 as 0.37 and 0.25 under 5 and 10 N, respectively, resulting in the lowest contact temperature under 10 N as 21.85 °C and the second lowest contact temperature under 5 N as 21.2 °C. The higher wear performance of G-5 is attributable to a variety of reasons in the light of experimental and theoretical data. As shown in Table 4, there is a ∼46% increase in the elastic modulus (*E_t_*) of the neat blend with 5 wt % GNP addition compared to the neat matrix, which can be held responsible for finer wear debris formation at this filler ratio due to the increased stiffness and toughness, thus a higher amount of worn material is retained on the surface via tribo-film formation. Indeed, the 2D wear profiles in Figure 10 show that the lowest overall wear depth belongs to G-5 sample surfaces. This situation is also reflected in the 3D profiler images with narrower ranges of color scale in Figure 14a,c. Aside from the GNP-driven effect of tribo-film formation, another contribution comes from the lower depth of maximum shear stress (*z*) and contact diameter (2a) values calculated for the G-5 sample respectively as 0.095 and 0.382 mm for 5 N; and respectively as 0.12 and 0.481 mm, for 10 N loading conditions (Table 4). As previously mentioned, these values can be regarded as multipliers in the theoretical calculation of worn volume; the contact area (2a) represents two dimensions, and the depth of the max. shear stress (*z*) represents the remaining dimension. Since G-5 sample also exhibited the highest un-notched Izod impact strength among all samples with 3361 kJ/m^2^, against 1833.1 kJ/m^2^ for the neat blend (Figure 5). The increased toughness of the blend composite is also accountable for the wear behavior taking place on the worn surface, as the wear proceeds in a tribo-film-oriented way which comprises the debris having the same mechanical properties of the surface. The tribo-film-oriented wear mechanism is also verified by the mentioned lumpy morphology of the surface decorated with signs of abrasive and adhesive wear such as detached and reattached wear debris and signs of plastic deformation (Figure 14a–d). As a result of the higher thickness and the higher extent of continuity of tribo-film layer as indicated by the lower 2D and 3D profile depths (Figures 10 and 14a–d), more of the wear phenomena took place on the compacted and sintered tribo-film layer which is bound with chemical bonding to a lesser extent than the bulk material, hence, a higher extent of abrasive and adhesive wear on the contact surface compared to the lower filler ratios. What is more, the gaps and pores around plastic deformation regions (Figure 14d) may be held accountable for the transfer of energy received by the PBT phase with poor toughness (Figure 5) to the PC constituent of the blend, hence the absorption of energy through plastic deformation.^5^

Figure 14e–h shows the SEM micrographs of G-7 contact surfaces worn under 5 and 10 N loading conditions. The extent of lumpy features observed on G-5 seems to be reduced under both loads, and the boundary of the wear tracks are less distinct specifically under 5 N, where the extensions of irregularities from nonworn regions can be observed on the wear track, which is a sign of a less continuous tribo-film formation. The reduction in the tribo-film formation can be inferred from ∼115% increase in the specific wear rate under 5 N from 7.4 × 10^–15^ m^3^(Nm)^−1^ for G-5 to 15.9 × 10^–15^ m^3^(Nm)^−1^ for G-7 and ∼30% increase in the specific wear rate under 10 N from 6.4 × 10^–15^ m^3^(Nm)^−1^ for G-5 to 8.3 × 10^–15^ m^3^(Nm)^−1^ for G-7 (Table 2); as also indicated by the 2D wear profiles in Figure 10, as well as the variation of the color scale in the inset 3D profiler images in Figure 18e,g. The reduction in tribo-film formation is also reflected in the average COF as a slight increase from 0.37 for G-5 to 0.42 for G-7 under 5 N, and from 0.25 for G-5 to 0.27 for G-7 under 10 N. The significant difference in the variation of specific wear rates under 5 and 10 N for G-5 and G-7 samples is attributable to the significant effect of contact pressure on the integrity of sintered tribo film; thus, under 10 N, such reduction is alleviated compared to 5 N. Localized lumpiness highlighted as more discernible plastic deformation areas compared to their surroundings in Figure 14e is another sign of the lesser extent of tribo-film formation compared to G-5, where plastic deformations decorated all contact regions (Figure 14b,d). Although to a limited extent, reduced tribo-film formation resulted in increased contact temperatures under both loads (from 21.2 to 21.55 °C under 5 N and from 21.85 to 22.00 °C under 10 N). The slight increase in the specific wear rate compared to G-5 is ascribed to the higher aptitude of formation of aggregates on the contact surface after a certain ratio of nanofiller weight within the composite as also reported in various studies on tribological properties of polymer composites prepared by melt-compounding.^36,68,70^ Impaired dispersion of nanofiller hinders the relative motion of the counter-bodies.^63^ This is also indicated by the slight decrease in the Shore-D hardness compared to G-5 (from 82.17.83 to 81.5 in Figure 11). When the abrasive ball sliding on the composite blend surface reaches GNP-agglomerated regions, more material is removed from the surface as compared to the regions with more homogeneous dispersion.^71^ Such impairment in the physical properties with increased filler ratio is attributed to the strong intermolecular bonding between GNPs that cannot be alleviated via melt-mixing^36^ after reduction of the space occupied by GNP particles^63^ and this behavior outcompetes the weak interaction between graphene and the polymer matrix. The previously mentioned viscoelastic behavior of the tribo film manifesting itself with macro-scale groove formations (or parallel waves indicated by the inset rectangular image indicator in Figure 14e) is also visible under 5 N and absent under 10 N, meaning that the contact pressure threshold required for the viscoelastic transition coincides with the contact pressure (65.5 MPa) resulting from 5 N load and exceeded by the contact pressure under 10 N load (82.5 MPa) for the G-7 surface (Table 4). It can be stated based on the experimental and calculated data that the friction and wear behavior of the neat and the GNP-filled PC–PBT blend nanocomposites was significantly affected by the presence of GNP, such that the lowest specific wear rate by G-5 (7.4 and 6.4 × 10^–15^ m^3^(Nm)^−1^) obtained respectively under 5 and 10 N are ∼86 and ∼90% lower than the highest specific wear rates exhibited by the neat PC–PBT blend, whereas they are ∼54% (compared to G-7 5 N) and ∼70% (compared to G-0.5 10 N) lower than those obtained from the most severely worn GNP-filled samples under 5 and 10 N, respectively. It is also worth noting that, in the specific wear rate graph (Figure 9), higher wear rates arose under higher load, except under G-5 and G-7. This is attributed to the more GNP-induced tribo-layer-oriented wear behavior exhibited by these samples, which is more susceptible to the increase in the contact pressure for compaction and integrity of the sintered contact region.

#### 3.3.3 SEM and EDS Analyses of Steel Ball Surfaces

The SEM images of the contact regions on the steel balls and the corresponding EDS-mapping analyses are shown in Figures 15 and 16 to elaborate the effect of the absence or presence of a transfer-film mechanism on the wear performance of the nanocomposite samples. The contact region on the abrasive ball used to wear the PC–PBT surface under a 10 N load is shown in Figures 15a and 19b. Parallel grooves in the high-magnification image in Figure 15b attest to direct contact between the steel ball and the neat sample surface, resulting in abrasive wear-induced scratches. The SEM micrographs as well as the EDS-mapping analysis results in Figure 15c show that the scratches are filled with a negligible amount of transfer material, which cannot be referred to as a film. On the other hand, the SEM images and the EDS-mapping results of G-0.5 sample shown in Figure 15d–f indicate that the contact region is decorated with a higher amount of transfer material, although to a limited extent. This is verified by the EDS analysis results (Figure 15f) where carbon-rich regions coincide with oxygen-rich regions as well as the regions void of the Fe atom, as opposed to the EDS results of the neat PC–PBT (Figure 15c) where carbon-smeared areas do not induce such alterations in oxygen and iron distribution. It can be consequently inferred that the direct contact between the counter-bodies is partly restricted by the addition of 0.5% GNP. The transfer material on the ball surface abrading the G-0.5 counter-body thus sufficed to prevent scratch formation which was observed on the neat sample’s sliding counterface (Figure 15).

**Figure 15:**
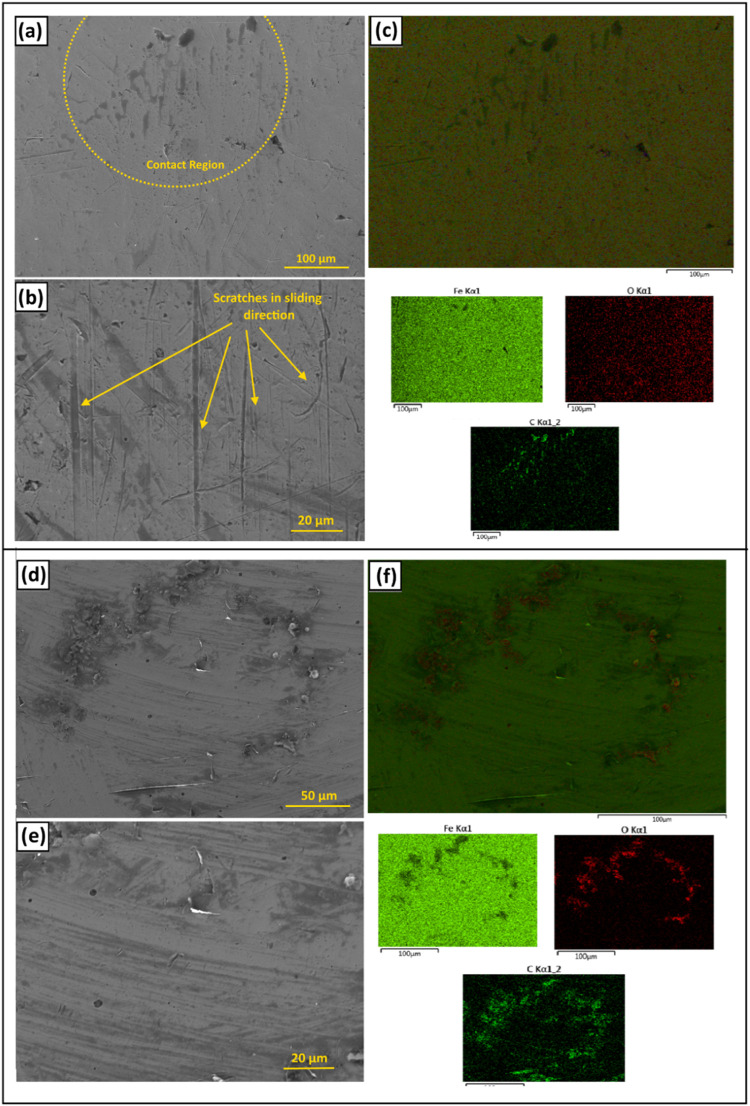
(a, d) Low magnification; (b, e) high-magnification counter surface SEM images; and (c, f) EDS-mapping analysis of the contact regions on the abrading balls for PC–PBT and G-0.5, respectively, under 10 N load.

The ball surface used to abrade the G-1 sample (Figure 16a–c) is also characterized by dark tones in random directions accompanied by few slight scratches indicating a limited degree of contact between the counter-bodies. Such scratches were likely induced by the increased moduli of the transfer film with each subsequent increase in the filler ratio (Table 4). The carbon atom distribution in the EDS analysis in Figure 16c verifies the presence of the transfer material in the darker regions. However, the amount of transfer film does not suffice to induce alterations on Fe and O atoms, implying that a transfer film was formed but could not be retained on the ball surface, which is also evident on the SEM micrographs of the steel ball surface (Figure 16a,b). Despite the increasing likelihood of transfer film formation with increasing GNP filler ratio, the extent of moduli and toughness did not suffice to form a consistent transfer layer at the contact region at these initial filler fractions.

**Figure 16:**
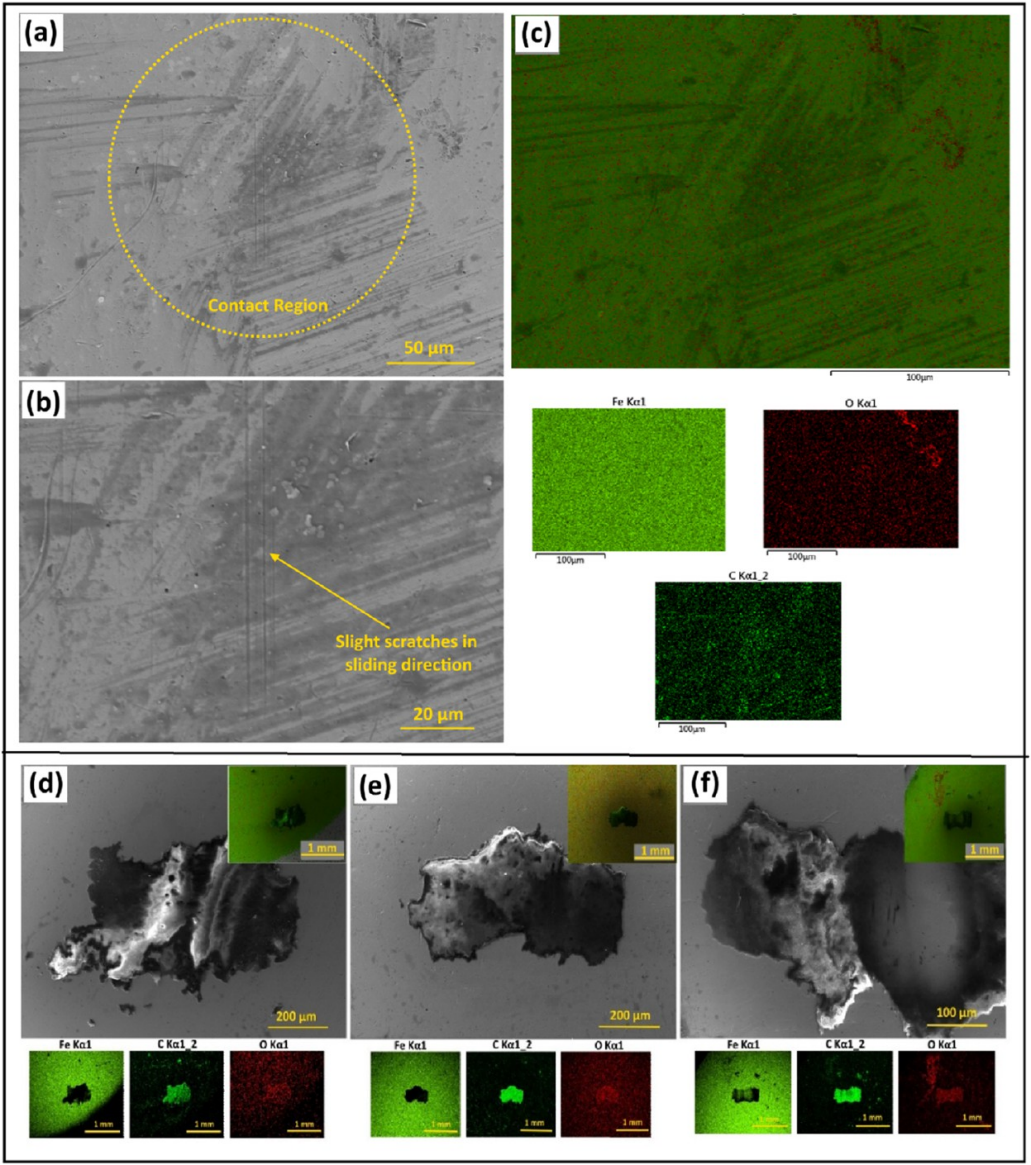
(a) High-magnification; (b) low magnification counter surface SEM images; (c) EDS-mapping analysis of the contact region on the ball for the G-1 sample; SEM images and EDS-mapping analyses of (d) G-3, (e) G-5, and (f) G-7 used under 10 N load.

Figure 16d–f show the contact areas on the ball surfaces sliding against samples G-3, G-5, and G-7 respectively. For all three filler ratios, formation of a transfer film and its retainment on the ball surface are evident with the distinct lumpy morphology (Figure 16d,e). The presence of a thick transfer layer is also verified by the overlapping of C atoms with Fe-free and O-rich areas in Figure 16f. The absence of a thick transfer layer on the ball surfaces of G-0.5 and G-1, and its presence on those of G-3, G-5, and G-7 indicates that the contributive effect of increasing filler content on transfer film formation became sufficient to sustain its presence on the ball surface. Retainment of a significant amount of transfer film on the ball surface as from the filler ratio of 3 wt % is consistent with the specific wear rate results (Figure 9) as well as the SEM discussions on wear tracks (Figures 12–14) implying a higher extent of transfer film-induced wear mechanism with increasing filler ratio.

## 4 Linear Regression and Machine Learning-Based Predictions

The machine learning algorithms were used to predict or forecast outcomes based on the experimental data. During the machine learning process, a model is trained using historical data to enable recognition of patterns and relationships, which are then utilized to predict future or unknown values. In this study, linear regression was applied as a statistical method to predict the tensile strength (*T*_s_), modulus (*E_t_*), and ductility (*e*_a_), whereas machine learning (ML) models (Decision Tree, Random Forest, Support Vector Regression (SVR), and Gradient Boosting) were utilized for prediction of the specific wear rates based on material properties and experimental data received from mechanical and tribological experiments. The machine learning models used in the present research were chosen on the basis of the nature and complexity of the target variables (mechanical and tribological properties), as well as the underlying physical phenomena. Linear regression was employed to predict the mechanical properties due to their more direct correlation with the filler content and limited number of input parameters.^73^ Specific wear rate, on the other hand, is affected by multiple nonlinear interactions (such as filler content, load, contact pressure, etc.) rendering machine learning (ML) algorithms such as Decision Tree (DT), Support Vector Regressor (SVR), Random Forest (RF) and Extreme Gradient Boosting (XGB) more suitable for this task, owing to their capability to capture nonlinear relationships thus ensuring an adequately accurate prediction of wear behavior, as also supported by recent literature.^74,75^

The analyses were performed using MATLAB, where each model was trained on the data set to establish correlations between input parameters, such as load, GNP ratio, and material properties, and the specific wear rate. Model performances were assessed using metrics such as *R* ^2^ (Coefficient of Determination), RMSE (Root Mean Squared Error), MSE (Mean Squared Error), and MAE (Mean Absolute Error). A summary of the approach and results for each model is presented, highlighting their effectiveness in the prediction of mechanical and tribological responses.

Performance metrics provide a numerical assessment of how well machine learning (ML) models fit the actual data. For supervised learning regression tasks, *R* ^2^ is a key performance metric. It represents the proportion of the variance in the data explained by the model. *R* ^2^ values range from 0 to 1, where *R* ^2^ = 0 indicates no correlation and the model fails to explain any variation in the data. An *R* ^2^ value of less than 0.5 indicates a weak correlation, and the model is considered inadequate for predicting the output. *R* ^2^ values between 0.7 and 0.9 signify satisfactory performance, while values greater than 0.9 suggest a highly effective model. An *R* ^2^ of 1 indicates a perfect fit, meaning the model explains all the variation in the data without error, which is rare for experimental data. MAE, MSE, and RMSE are additional key metrics for evaluating model performance. MAE measures the average of the absolute differences between predicted and actual values. MSE calculates the average of the squared differences between actual and predicted values, while RMSE is the square root of MSE. These error metrics provide valuable insight into the model’s performance. Smaller values of these errors indicate higher accuracy in the model’s predictions.^76^ Aydin et al. (2023) explain utilizing five supervised machine learning algorithms to predict the wear performance of ZK60/CeO2 composites. They used the Support Vector Regressor (SVR), Random Forest (RF), Multi-Layer Perceptron (MLP), Extreme Gradient Boosting (XGB), and Decision Tree (DT) models. In this work, the decision tree model was employed using the CART (Classification Regression Tree) algorithm via the fitrtree function of MATLAB. CART implements binary recursive partitioning and variance minimization for continuous variable prediction^77^ rendering it a suitable choice for the wear rate data set used in this study.

The ML models were trained on experimental data, which included parameters such as sliding speed, filler content, and load, to predict volume loss (wear performance). The models’ predictions were evaluated based on metrics such as *R* ^2^, RMSE, MSE, and MAE. The study concluded that tree-based methods (DT, RF, XGB) showed superior predictive performance compared to nontree-based models (SVR, MLP).^78^ In the data preprocessing step, no explicit noise filtering was employed, as all features were obtained from controlled experimental procedures and averaged over multiple repetitions. Feature selection was employed manually based on domain knowledge including material properties, tribological parameters, and calculated contact mechanics data to ensure physical interpretability and relevance to wear data. The data sets for all machine learning models consist of 6 × 2 = 12 combinations of independent input variables (6 GNP weight fractions as 0, 0.5, 1, 3, 5, and 7 wt % and two loads as 5 and 10 N) for which dependent input variables (*E_t_*, *P*_max_, τ_max_, *z* and 2a) shown in Table 4 acquired from the tensile tests and Hertzian contact pressure calculations were used.

### 4.1 Linear Regression Model

The most common types of regression analyses are Linear regression, Logistic regression, and Cox regression. Linear regression is a key tool in statistical analysis. Its wide range of applications include describing relationships, making estimations, and forecasting.^79^

Linear regression is a statistical technique used to model the relationship between a dependent variable and one or more independent variables by fitting a linear equation to the observed data. Figure 17 (tensile strength), Figure 18 (modulus), and Figure 19 (ductility) show the analysis of test data for three different material properties (modulus, tensile strength, and ductility) using simple linear regression. First, linear regression models are created for each property, and predictions are made. Subsequently, error metrics, such as *R* ^2^, RMSE, MSE, and MAE are calculated to evaluate the accuracy of the model. Then, a two-subplot visualization is generated, comparing the actual and predicted values for each property, along with the regression line. This visualization also includes an error plot, and the calculated metrics are displayed on the graph. These graphs show the comparison of actual versus predicted values for tensile strength (*T*_s_), modulus (*E_t_*), and ductility (*e*_a_) based on linear regression models. In each graph, the regression lines closely match the actual data, indicating a relatively good fit, as reflected in the high *R* ^2^ values, especially for ductility (*R* ^2^ = 0.9267), tensile strength (*R* ^2^ = 0.8419) and modulus (*R* ^2^ = 0.8490).

**Figure 17:**
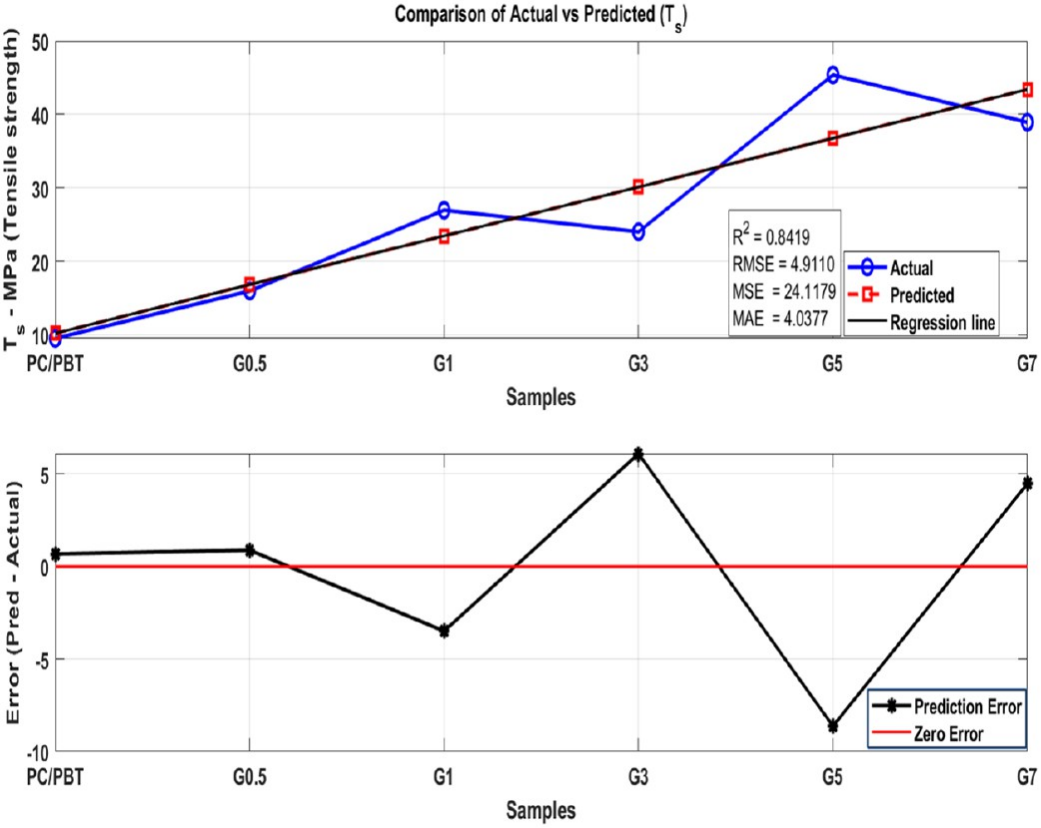
Comparison of actual vs predicted tensile strength (*T*_s_) and prediction error.

**Figure 18:**
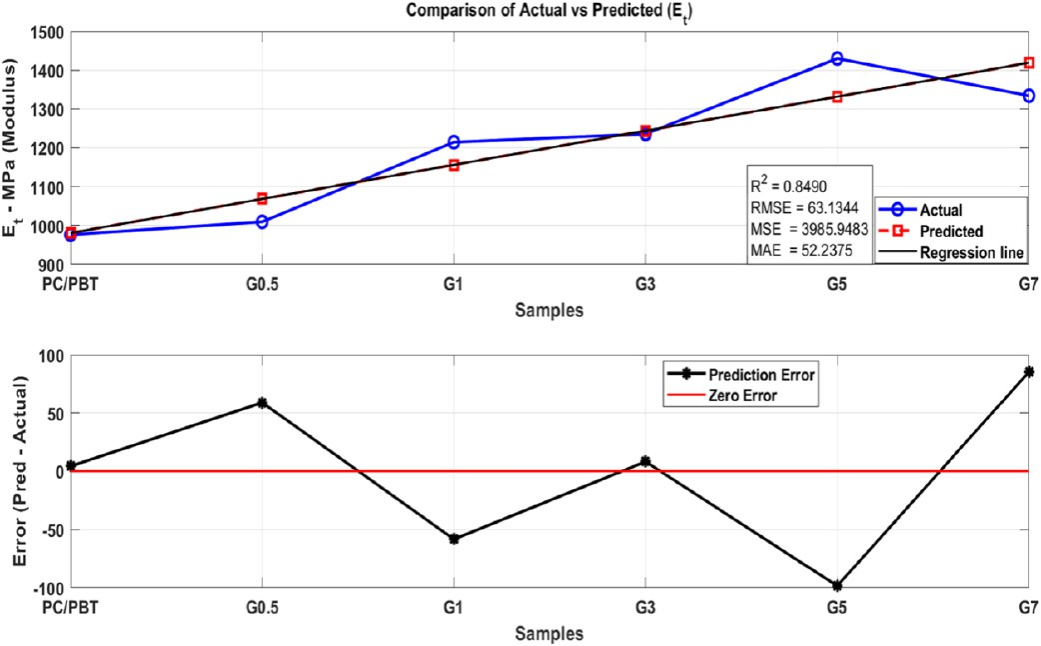
Comparison of actual vs predicted modulus (*E_t_*) and prediction error.

**Figure 19:**
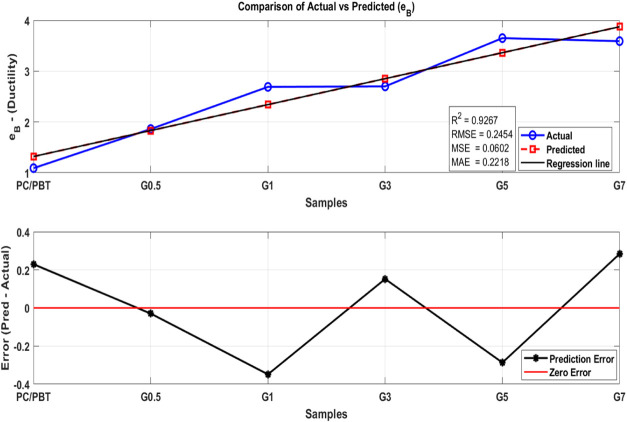
Comparison of actual vs predicted ductility (*e*_a_) and prediction error.

### 4.2 Decision Tree (DT) Model

Decision Tree models have been employed as machine learning models for both regression and classification tasks. This model works by creating a tree-like structure that makes decisions based on input features, splitting the data into subsets via effective separation of data, allowing the model to predict the outcomes or to classify data points based on the patterns it has learned. A decision tree model is created in MATLAB using material properties and test data, and this model is used to predict the wear rate. First, a data set is created, and the decision tree model is trained, followed by evaluation of model performance using metrics such as *R* ^2^, RMSE, MSE, and MAE. Finally, the graph in Figure 20 comparing the actual and predicted wear rates is provided. The graph shows a comparison between the actual and predicted wear rates using a decision tree model with the predicted values closely following the actual data. The high *R* ^2^ value of 0.9581 indicates that the model provides a strong fit, while the error metrics (RMSE, MSE, and MAE) signify a relatively low prediction error.

**Figure 20:**
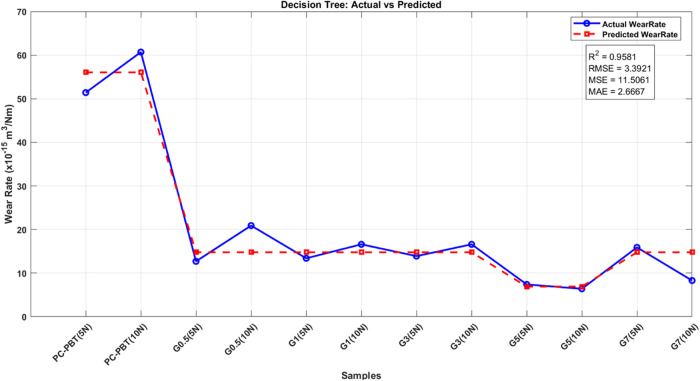
Decision Tree: actual vs predicted wear rate.

### 4.3 Random Forest (RM) Model

Random Forest is a machine learning algorithm that primarily uses classification and regression trees and is a flexible, easy-to-use, and efficient method often producing large results. The RF algorithm belongs to the group of ensemble learning algorithms based on the bagging method. One of the key advantages of Random Forest is its ability to provide solutions for both classification and regression problems, which form the foundation of other machine learning algorithms. The decision trees that make up the Random Forest are built in parallel, and these trees can consist of both classification trees and regression trees. The nodes in each decision tree are split using the best attributes that can provide the optimal solution among all features^80^

Figure 21 compares the actual and predicted wear rates using a random forest model. The predicted values (red squares) highly match the actual values (blue circles), but a discrepancy can be noticed at the beginning of the data, especially for the first few samples. Overall, the model performs well for most of the samples, as indicated by the high consistency between the actual and predicted wear rates, although some prediction errors can be observed at certain points. In this MATLAB code, a Random Forest regression model was used to predict wear rates based on the material properties and experimental data, and the model is created using the fitrensemble function with bagging, consisting of 50 learning cycles, where each decision tree is trained with a minimum leaf size of 2. After training the model, predictions are made on the training data, and performance metrics like *R* ^2^ (0.8382), RMSE (6.6666), MSE (44.4442), and MAE (4.9023) are calculated to assess the model’s accuracy. Finally, a graph is generated by comparing the actual and predicted wear rates, showing how closely the predicted values match the actual ones, with a legend and grid for ease of interpretation.

**Figure 21:**
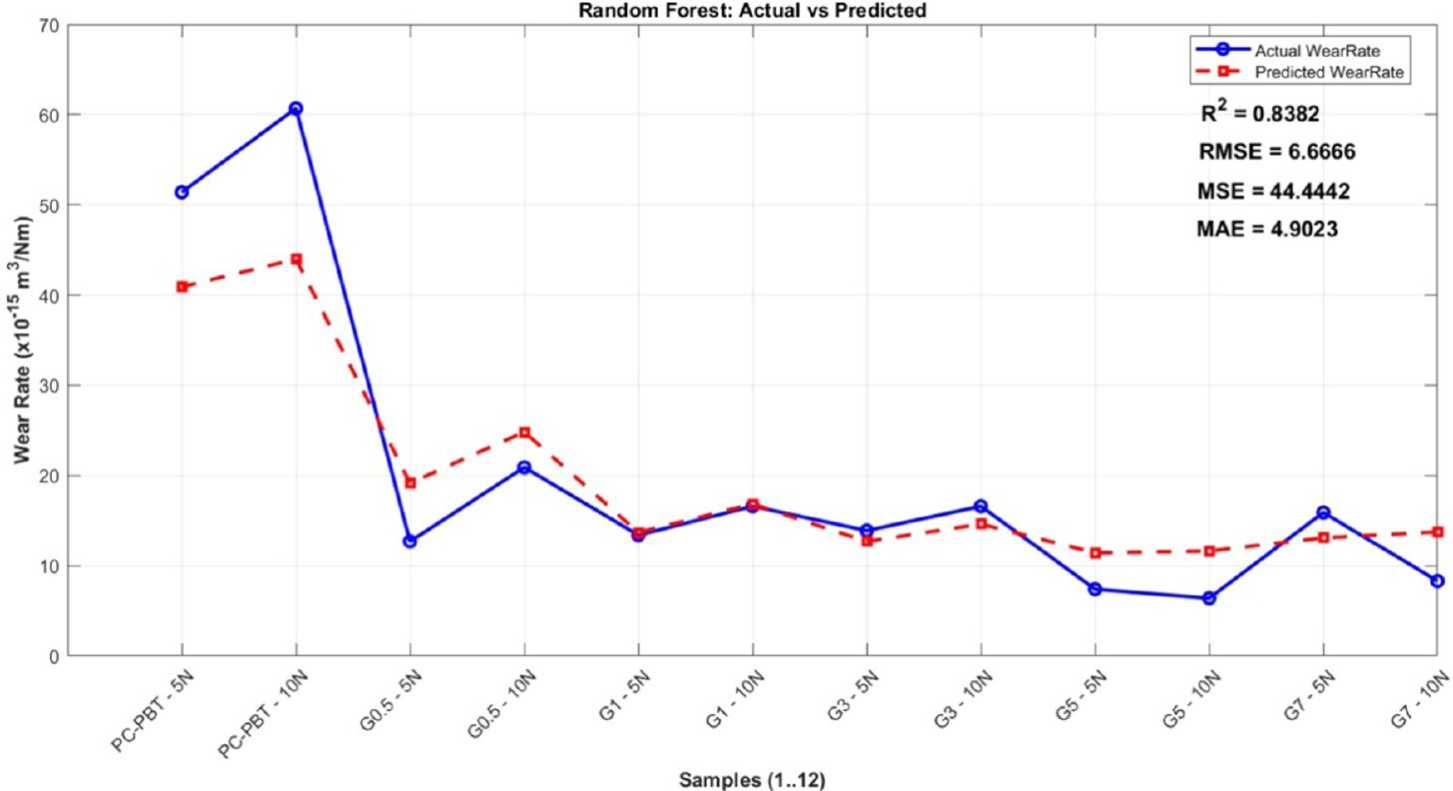
Random Forest: actual vs predicted wear rate.

### 4.4 Support Vector Regression (SVR) Model

Support Vector Regression (SVR) is a machine learning model that employs support vector machines to evaluate continuous values by finding a line or hyperplane that best fits the data while keeping prediction errors as small as possible within a set margin. Smola and Schölkopf (2004) introduced a comprehensive guide to Support Vector Regression (SVR), covering its basic principles, algorithms, and recent enhancements. It explains the theory underlying SVR, optimization techniques, and how SVR is employed for regression problems. The guide also discusses key ideas like kernels, dual problems, and cost functions and explores more advanced topics such as feature mapping and choosing the right kernel. Additionally, it emphasizes the importance of regularization, SVR’s ability to cope with nonlinear regression, and the methods to enhance SVR performance in real-world applications^81^

The MATLAB code employed in the current work performs a manual grid search for Support Vector Regression (SVR) to predict wear rates based on material properties and experimental data. The process involves defining a range of parameters (BoxConstraint, Epsilon, and KernelScale), and evaluating different combinations using cross-validation to find the model with the highest *R* ^2^ value. The best-performing model is then used to make final predictions on the entire data set, and performance metrics such as *R* ^2^, RMSE, MSE, and MAE are calculated and displayed. Finally, an “Actual vs Predicted” graph is plotted to visualize the comparison between the model’s prediction results with the actual wear rates. Figure 22 displays the comparison between the actual and predicted wear rates by using the Support Vector Regression (SVR) model after a grid search. The predicted values (red squares) closely match the actual values (blue circles), signifying strong model performance. The high *R* ^2^ value of 0.9865 implies that the model explains most of the variance in the wear rates, while the error metrics (RMSE = 1.9268, MSE = 3.7127, and MAE = 1.8932) indicate relatively low prediction errors, demonstrating good predictive accuracy.

**Figure 22:**
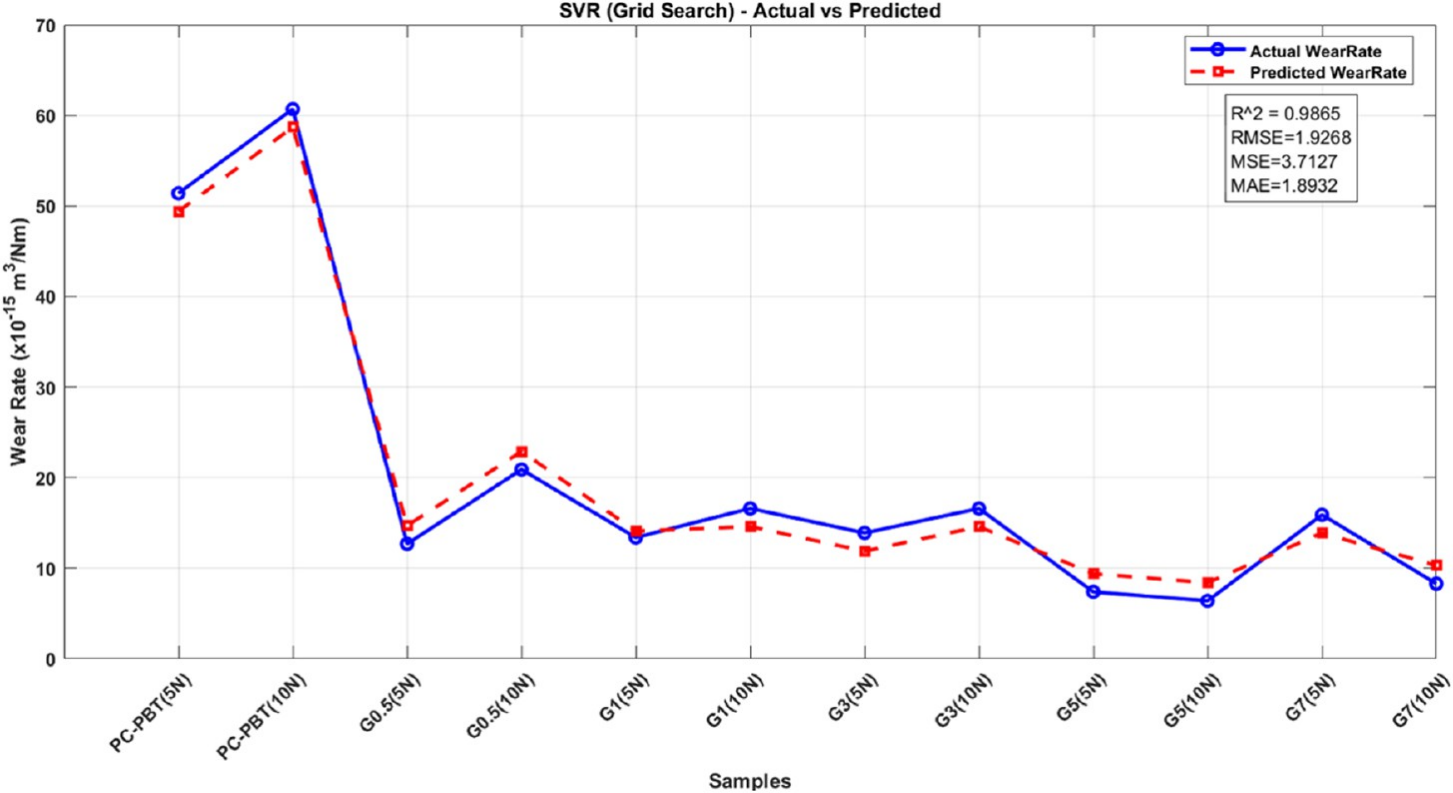
SVR model: actual vs predicted wear rate.

### 4.5 Least-Squares Boosting (LSBoost) Model

Gradient boosting regression tree algorithms use an ensemble learning approach, where strong forecasting models are created by combining multiple individual regression trees (decision trees), which are known as weak learners.^82^

LSBoost (Least-Squares Boosting) is a machine learning technique based on the gradient boosting framework. It is a variant of boosting algorithms minimizing the least-squares error by iteratively fitting weak models, usually decision trees, to the residuals of the predictions from the previous models. In LSBoost, each new model is trained for the correction of the errors (or residuals) of the previous models, and the predictions are combined to form the final output. Unlike traditional boosting, which focuses on minimization of an arbitrary loss function, LSBoost specifically uses a least-squares loss (or mean squared error, MSE) as the objective. This renders it particularly effective for regression tasks. In MATLAB, the fitrensemble function can be used to employ LSBoost, where the user can specify the number of learning cycles (trees), learning rate, and other parameters such as the minimum leaf size for the decision trees. This MATLAB code utilizes a gradient boosting model (LSBoost, similar to Extreme Gradient Boosting (XGBoost)) to predict wear rates based on material properties and experimental data. The process involves preparation of a feature matrix (*X*) and a target variable (*Y*), then training the model using 30 learning cycles, a learning rate of 0.2, and a minimum leaf size of 2 to prevent overly deep trees. After training the model, predictions are made, and performance metrics such as *R* ^2^, RMSE, MSE, and MAE are calculated to assess the model’s accuracy. Finally, a plot is generated for comparison of the actual and predicted wear rates, displaying the *R* ^2^ and other performance metrics in a text box on the graph. Figure 23 compares the actual and predicted wear rates by using the LSBoost model. The predicted values (red squares) closely follow the actual values (blue circles), indicating a strong model fit. The high *R* ^2^ value of 0.9922 shows that the model explains a large portion of the variance in the wear rates. The error metrics, including RMSE (1.4631), MSE (2.1407), and MAE (1.2113), suggest that the model performs with a relatively low error, indicating good predictive accuracy. Overall, the LSBoost model appears to be highly effective in predicting wear rates with high precision.

**Figure 23:**
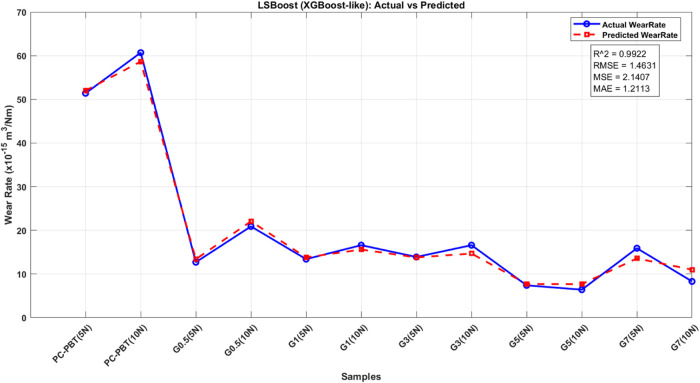
LSBoost model: actual vs predicted wear rate.

Table 5 presents the performance metrics of the machine learning models employed to predict the specific wear rate data by using the load and the GNP filler ratio as independent input variables and the calculated contact properties provided in Table 4 as the dependent input variables. As indicated by the performance metrics the most accurate predictions are made by the LSBoost model achieving a *R* ^2^ value of 0.9922 and the least accurate performance is demonstrated by the RM model with a *R* ^2^ value of 0.8503. The high prediction capability of DT, SVR and LSBoost models can be ascribed to their capability to incorporate the effect of dependent variables into the model.

**Table 5:** Performance Metrics of Machine Learning Models

	*R* ^2^	RMSE	MSE	MAE
DT	0.9581	3.3921	11.5061	2.6667
RM	0.8382	6.6666	44.4442	4.9023
SVR	0.9865	1.9268	3.7127	1.8932
LSBoost	0.9922	1.4631	2.1407	1.2113

## 5 Conclusions

In the present work, the mechanical and tribological properties of PC–PBT blend composites reinforced with varying filler weight fractions of GNP were investigated. The following conclusions can be drawn from the theoretical and experimental results:

- Increasing GNP fraction resulted in increased stiffness, indicated by ∼46% increase in tensile modulus (*E_t_*) and ∼38% increase in flexural modulus (*E*_f_) for G-5 sample compared to the neat blend. Likewise, tensile and elastic ductility increased by ∼234 and ∼287%, respectively and the impact strength was improved by ∼78% with the optimum filler fraction of 5 wt %, due to the increased crystallinity of the PC–PBT blend with increasing GNP content attributed to suppressed transesterification.
- Sample with 5 wt % fraction (G-5) exhibited 27 and 24% reduction in average COF under 5 and 10 N, respectively compared to the neat sample. Moreover, G-5 yielded an outstanding reduction in specific wear rates, such that ∼90 and ∼86% reduction was achieved under 10 and 5 N, respectively. The factors contributing to this achievement can be summarized as follows:

- Increased stiffness with increasing filler fraction, which led to reduction in the calculated depths of max. shear stress (*z*) and the contact area diameters (2a), which can be deemed as 1-dimensional and 2-dimensional theoretical multipliers for volumetric wear loss.
- Lubricating effect of GNP layers with improved interlayer shearing.
- Reduction in the contact temperatures of composite samples compared to the neat sample, attesting to increased heat dissipation due to the high thermal conductivity of GNPs.
- Increased extent of tribo-layer-oriented wear regime with increasing nanofiller content.
- After the filler ratio of 3 wt % significant amount of transfer film is retained on the ball surface, preventing the direct contact between the counter-bodies, thus reducing the friction and wear loss.

- Highly accurate performance metrics with up to *R* ^2^ = 0.9922 (with LSBoost model) were achieved for the prediction of specific wear rates by machine learning models due to the models’ capability to incorporate the effect of dependent variables from contact calculations into specific wear rate estimations.

The significantly better wear performance of PC–PBT blend composites achieved by the addition of GNPs implies that further improvement in wear and friction behavior is possible via application of toughening routes for PC–PBT blends prior to incorporation of GNP particles, which warrants further research on the subject.

## Data Availability

The data that support the findings of this study are available throughout the manuscript.
